# Enhanced replication fitness of MERS-CoV clade B over clade A strains in camelids explains the dominance of clade B strains in the Arabian Peninsula

**DOI:** 10.1080/22221751.2021.2019559

**Published:** 2021-12-17

**Authors:** Nigeer Te, Jordi Rodon, Mónica Pérez, Joaquim Segalés, Júlia Vergara-Alert, Albert Bensaid

**Affiliations:** a IRTA, Centre de Recerca en Sanitat Animal (CReSA, IRTA-UAB), Bellaterra, Cerdanyola del Vallès, Spain; b UAB, CReSA (IRTA-UAB), Bellaterra, Cerdanyola del Vallès, Spain; c Departament de Sanitat i Anatomia Animals, Facultat de Veterinaria, UAB, Bellaterra, Cerdanyola del Vallès, Spain

**Keywords:** Alpaca, IFN, Middle East respiratory syndrome coronavirus, MERS-CoV, orf4a, strain

## Abstract

Middle East respiratory syndrome coronavirus (MERS-CoV) continues infecting humans and dromedary camels. While MERS-CoV strains from the Middle East region are subdivided into two clades (A and B), all the contemporary epidemic viruses belong to clade B. Thus, MERS-CoV clade B strains may display adaptive advantages over clade A in humans and/or reservoir hosts. To test this hypothesis *in vivo*, we compared an early epidemic clade A strain (EMC/2012) with a clade B strain (Jordan-1/2015) in an alpaca model monitoring virological and immunological parameters. Further, the Jordan-1/2015 strain has a partial amino acid (aa) deletion in the double-stranded (ds) RNA binding motif of the open reading frame ORF4a protein. Animals inoculated with the Jordan-1/2015 variant had higher MERS-CoV replicative capabilities in the respiratory tract and larger nasal viral shedding. In the nasal mucosa, the Jordan-1/2015 strain caused an early IFN response, suggesting a role for ORF4a as a moderate IFN antagonist *in vivo*. However, both strains elicited maximal transcription of antiviral interferon-stimulated genes (ISGs) at the peak of infection on 2 days post inoculation, correlating with subsequent decreases in tissular viral loads. Genome alignment analysis revealed several clade B-specific amino acid substitutions occurring in the replicase and the S proteins, which could explain a better adaptation of clade B strains in camelid hosts. Differences in replication and shedding reported herein indicate a better fitness and transmission capability of MERS-CoV clade B strains than their clade A counterparts.

## Introduction

Middle East respiratory syndrome coronavirus (MERS-CoV) causes a severe respiratory disease in humans. The Arabian Peninsula continues to be the global epicenter of the disease [1,2]. As of November 2021, the World Health Organization (WHO) reported 2,578 infections and 888 fatalities (∼34.4%) in 27 countries [3]. While most cases have been documented in The Kingdom of Saudi Arabia, a major travel-associated outbreak in South Korea reminds that MERS-CoV is of global concern [4,5]. Due to negative impacts on public health and the economy, MERS-CoV is listed as one of the WHO Research and Development Blueprint priority pathogens.

So far, the dromedary camel is the only confirmed source of zoonotic infection, while new world camelids (alpacas and llamas) are also susceptible to MERS-CoV under both natural and experimental conditions [6–11]. The human acquisition is primarily due to close contact with infected camels [12]. Although limited human-to-human transmission has been reported within families, contagion mostly occurred in health-care settings [13].

MERS-CoV is phylogenetically divided into three major clades, temporarily named clade A, B and C [14–16]. Sequence examination of various MERS-CoV strains revealed that nowadays only clade B viruses are circulating in the Arabian Peninsula and have replaced clade A in humans and dromedary camels [16–18]. Therefore, clade B viruses might possess an evolutional advantage against clade A isolates, exhibiting enhanced replicative fitness when infecting human/dromedary hosts. However, data characterizing phenotypic and pathogenic differences between these two clades of MERS-CoV are scarce. Recently, Wang et al. [19] showed that a clade B virus (ChinaGD01) led to more weight loss, severe lung lesions and higher viral titres in the lung of human dipeptidyl peptidase-4 (h-DPP4) transduced mice than did the prototype virus EMC/2012 (clade A). However, in a previous study, apparent contradictory results were reported since clade A and B viruses exhibited comparable lung titres and pathology in h-DPP4 transduced mice [14]. Also, there was no significant difference in replication competence of these two clade viruses in human bronchial and lung explants [20].

Despite its role in antagonizing interferon (IFN) defense mechanisms [21–23], the accessory protein ORF4b is probably not implicated in virus replication *in vivo* [14]. Rather, non-structural proteins (nsp), which also interfere with IFN production [24,25], are thought to play a more important role in virus replication than accessory proteins. In that respect, clade B-specific mutations have been described in ORF1ab [19]. Also, the role of ORF4a as an IFN antagonist has not been assessed *in vivo* but mainly determined under *in vitro* conditions [21,26–28]. A recombinant virus lacking ORF4a showed increased expression levels of type I and III IFNs and slightly decreased replication in human airway epithelium-derived A549 cells [27]. Nevertheless, whether deletions/mutations of these viral factors lead to increased zoonotic potential of clade B strains lacks direct proof in reservoir hosts.

Recently, we characterized the pathogenesis of a clade B MERS-CoV strain (Qatar15/2015) in an alpaca model [29]. Upon MERS-CoV infection, few infected nasal epithelial cells could be detected by immunohistochemistry (IHC) as soon as 1-day post inoculation (dpi). Nasal tissues were maximally infected on 2 dpi and infection was extended to the bronchus, where some infected epithelial cells were observed. On the following days, the virus was gradually cleared. Alpacas responded at the peak of MERS-CoV infection with early and transient type I and III IFN responses detected exclusively in the nasal mucosa, concomitant with an induction of antiviral ISGs along the whole respiratory tract. Exacerbated inflammation was not observed in these animals, which displayed attenuated NF-κB and NLRP3 signalling cascades in conjunction with increased mRNA levels of the anti-inflammatory cytokines IL10 and type III IFNs.

To assess differences in virulence, replication and pathogenesis explaining field observations that MERS-CoV clade B strains have replaced clade A in camelids, we selected an early epidemic clade A isolate, EMC/2012 [30], and a clade B strain, Jordan-1/2015 [31], to experimentally infect alpacas. The Jordan-1/2015 strain harbours a 16 amino acid (aa) deletion in ORF4a suspected to affect the recognition of dsRNA by host RNA sensors [31]. Therefore, this experimental set up also allowed to gain insights on the role of ORF4a in MERS-CoV pathogenesis. Alpacas inoculated with Jordan-1/2015 exhibited higher MERS-CoV loads in the respiratory tract and larger viral shedding, demonstrating the increased replicative fitness of clade B strains in a camelid host. In addition, mRNA expression of cytokines and chemokines along the respiratory tract was monitored for both strains during three consecutive days. Thus, together with previous results obtained using the Qatar15/2015 isolate [29], a rational explanation is suggested for the predominance of MERS-CoV clade B strains in the Arabian Peninsula.

## Materials and methods

### Ethics statement

All animal experiments were approved by the Ethical and Animal Welfare Committee of IRTA (CEEA-IRTA) and by the Ethical Commission of Animal Experimentation of the Autonomous Government of Catalonia (file N° FUE-2018-00884575 – Project N°10370). The work with infectious MERS-CoV was performed in the biosafety level-3 facilities (BSL-3) of the Biocontainment Unit of IRTA-CReSA in Barcelona, Spain.

### Cell culture and virus

MERS-CoV EMC/2012 (GenBank Accession JX869059) and Jordan-1/2015 (GenBank Accession KU233364) isolates were propagated in Vero E6 cells and titrated, as previously described [10]. Viral stocks used for experimental infection procedures were provided by Dr Bart Haagmans from the Erasmus Medical Center (Rotterdam, The Netherlands) and were sequenced prior shipment to IRTA-CReSA. No mutations were found and virus was not propagated before inoculation to alpacas.

### Animal study and experimental design

Twenty-one 6-8-month-old alpacas (*Vicugna pacos*) were purchased and randomly assigned a number (AP1-AP21). AP19-AP21 were kept as non-infected controls and euthanized upon arrival. The remaining animals were acclimated for one week at the animal BSL-3 facilities. Alpacas, AP1-AP9 (group 1) and AP10-AP18 (group 2) were housed in two different pens and intranasally inoculated with a 10 [7] TCID_50_ dose of the MERS-CoV EMC/2012 and Jordan-1/2015 strains respectively, as described previously [10]. Three animals from each group were euthanized per day with an overdose of pentobarbital followed by exsanguination on 1, 2, and 3 dpi. Clinical signs were monitored and nasal swab (NS) samples were collected on the day of euthanasia for MERS-CoV titration and RNA detection, as previously described [10]. Complete necropsies were also performed. Collected respiratory tissues included: nasal turbinates (frontal, medial and caudal), large and small bronchus, and lung parenchyma (frontal, medial and caudal). Respiratory tissues were immersed in media for viral RNA detection by RT-qPCR; or fixed in formalin or methacarn for immunohistochemistry [10] and cytokine quantification respectively [29].

### Viral genomic and subgenomic RNA detection by RT-qPCR

NS and tissue samples were processed as previously described [10]. Following euthanasia, each respiratory tract tissue was cut into 0.5 × 0.5 cm pieces (weighing approximately 200–300 mg) per each sample at different dpi and homogenized using a TissueLyser II (Qiagen, Germany). Viral RNA from both NS and tissue homogenates was extracted using a NucleoSpin® RNA virus kit (Macherey-Nagel) following the manufacturer’s instructions. Genomic and subgenomic MERS-CoV RNA was quantified by the UpE [32] and M mRNA [33] RT-qPCR assays, respectively. Samples with a cycle threshold of less than 40 were considered positive.

### Virus isolation from NS

NS samples collected were evaluated for the presence of infectious virus at various dpi by titration in Vero E6 cells, as previously reported [10]. The amount of infectious virus in each sample was calculated by determining the TCID_50_/mL.

### Histopathology and immunohistochemistry

Respiratory tissue samples for pathological studies were fixed by immersion in 10% neutral-buffered formalin, embedded in paraffin, sectioned at 2.5 µm for slides and stained with haematoxylin and eosin. A monoclonal mouse anti-MERS-CoV N protein antibody (Sino Biological Inc., Beijing, China) was used to detect the presence of MERS-CoV antigen, as described before [10,34].

### Methacarn-fixed, paraffin-embedded (MFPE) tissue specimens

For the assessment of cytokine mRNA profiles, nasal turbinates, trachea and lung were fixed by immersion in methacarn and paraffin embedded. MFPE-tracheal and lung samples were processed for RNA isolation by scraping the whole section from the prepared slides and referred to as “scraped” tissues. MFPE-nasal specimens were processed for laser capture microdissection (LCM) prior to RNA extraction. All these procedures were previously established [29].

### LCM

LCM was used to micro-dissect IHC positive and negative nasal epithelia areas from the same tissue section, as previously described [29]. Briefly, four consecutive sections from the same MFPE-block containing nasal specimens were cut. One of the sections was subjected to IHC to localize infected/non-infected cells in the tissues and served as a reference (template) for the three subsequent sections which were stained with cresyl violet and then subjected to LCM (Supplementary Figure 1).

### Total RNA isolation and cDNA synthesis

Total RNA from micro-dissected or scraped tissues was extracted, and converted into cDNA synthesis, following previous standard protocols [29].

### Relative quantification of cytokines mRNA and viral genomic and subgenomic loads on MFPE tissues

A microfluidic RT-qPCR assay was used to quantify gene expression from the MFPE samples. The selection of innate immune genes and specific pair of primers to amplify their transcriptional products (Supplementary Table S1) were described in a previous work [29]. In addition, specific primers for the genomic (UpE) and subgenomic (M mRNA) viral regions were added to the assay [29]. Data were analysed with the software 4.1.3 (Fluidigm Corporation, South San Francisco, USA) and the DAG expression software 1.0.5. 6, as previously described [29]. The up-or down-regulated expression of each cytokine gene was expressed in fold change (Fc), all obtained values are available in the Supplementary Data S1. The quantification of MERS-CoV genomic (UpE) and subgenomic (M mRNA) RNA, obtained with the microfluidic qPCR assay, expressed in Cq values, are also available in the Supplementary Data S1.

### Statistical analysis

To use the Student’s *t*-test, a logarithmic 10 transformations were applied on Fc values to approach a log-normal distribution. Thus, the means of the logarithmic Fc obtained at different dpi for each group of animals could be compared. Significant upregulation or downregulation of genes was considered if they met the criteria of a relative Fc of ≥2-fold or ≤2-fold respectively with *P* < 0.05. The Shapiro-Wilk normality test was used to determine the normal distribution of RT-qPCR data (Cq values), prior to applying Tukey's multiple comparisons test to compare Cq values of tissue samples at different dpi. Differences were considered significant at *P* < 0.05. Correlation coefficients were determined using Spearman’s correlation test.

### Alignment of MERS-CoV proteins from clade A and B strains

The aa sequences from twenty-one MERS-CoV strains encompassing seven lineages (including the EMC/2012 and the Jordan-1/2015 strains) were retrieved from the NCBI database and aligned using the UniProt alignment tool (https://www.uniprot.org/align/). Details including the accession number of the strains are provided in Table 1.

**Table 1.** Information of the different MERS-CoV strains used for amino acid sequence alignment.

Name of strain	Origin	Clade	Lineage	Accession Number (NCBI)
EMC/2012	Human	A	1	JX869059
Jordan-N3/2012	Human	A	1	KC776174
KSA_CAMEL-376	Dromedary camel	B	1	KJ713299
Camel/UAE/D1209/2014	Dromedary camel	B	1	KP719933
Abu Dhabi_UAE_18_2014	Human	B	1	KP209307
Al-Hasa25/2013	Human	B	2	KJ156866
Riyadh_2_2012	Human	B	2	KF600652
England-KSA/1/2018	Human	B	3	MH822886
Camel/Riyadh/Ry23N/2014	Dromedary camel	B	3	KT368825
Makkah_C9355/KSA/Makkah/2014-04-15	Human	B	4	KM027261
KSA-CAMEL-505	Dromedary camel	B	4	KJ713295
Hu/UAE_032_2014	Human	B	4	KY581693
Jeddah/D90/2014	Dromedary camel	B	4	KT368844
Qatar15/2015	Human	B	5	MK280984
Jordan-1/2015	Human	B	5	KU233364
ChinaGD01	Human	B	5	KT006149
Riyadh_1_2012	Human	B	6	KF600612
Bisha_1_2012	Human	B	6	KF600620
Camel/UAE_B8_2015	Dromedary camel	B	7	MF598600
D2731.3/14	Dromedary camel	B	7	KT751244
Camel/UAE_B65_2015	Dromedary camel	B	7	MF598655

### Modelling of MERS-CoV PLpro and S proteins

The crystal structures of MERS-CoV PLpro and S proteins were obtained from the PDB (PDB ID: 4REZ for PLpro and PDB ID:5W9H for the S protein). Mutation sites were mapped onto structures using PyMOL (PyMOL molecular graphics system, version 2.5.0; Schrödinger, LLC).

## Results

### Clinical signs

To compare the pathology induced by strains EMC/2012 (clade A) and Jordan-1/2015 (clade B) two groups of alpacas, defined as groups 1 and 2 respectively, were intranasally inoculated with each strain. Three animals from each group were sacrificed per day and during three consecutive days. This timing was based on previous experiments performed in alpacas with the EMC/2012 [6] and Qatar15/2015 (clade B) [29] strains where maximal viral shedding loads in tissues were observed at 2 dpi. None of the animals showed clinical signs and basal body temperatures remained normal (below 39.5°C) in response to both MERS-CoV strains throughout the study.

### Nasal viral shedding of alpacas upon MERS-CoV challenge

**Figure 1.**
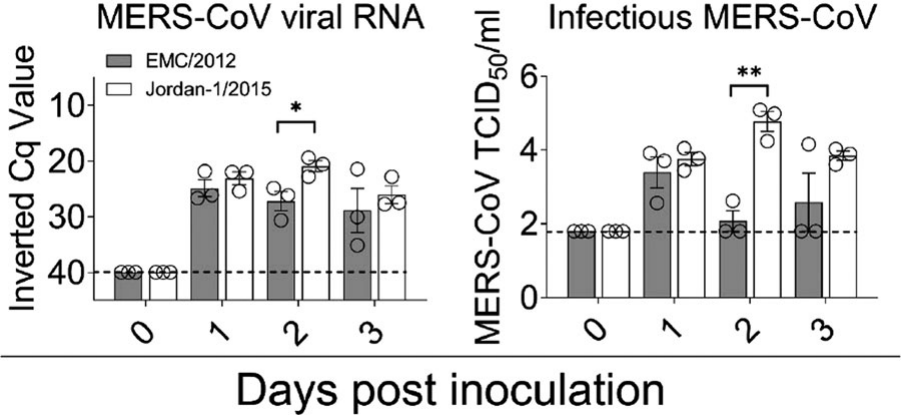
Viral loads in nasal swabs (NS) of MERS-CoV EMC/2012 and Jordan-1/2015 infected alpacas. Viral RNA (left) and infectious MERS-CoV (right) loads from NS samples collected at different time points. Viral RNA shedding was determined by the UpE RT-qPCR assay. Each bar represents the mean Cq value ± SEM of NS from 3 animals euthanized on 0, 1, 2 and 3 dpi, respectively. Statistical significance was determined by Tukey's multiple comparisons test. *P < 0.05; **P < 0.01; ***P < 0.001; ****P < 0.0001. Dashed lines depict the detection limit of the assays. Abbreviations: Cq, quantification cycle; MERS-CoV, Middle East respiratory syndrome coronavirus; TCID_50_, 50% tissue culture infective dose.

NS were obtained from all animals on the day of euthanasia (0, 1, 2 and 3 dpi). All MERS-CoV inoculated alpacas shed viral RNA at all time points. At 2 dpi, Cq values of NS were statistically lower for alpacas inoculated with the Jordan-1/2015 strain when compared to those challenged with the EMC/2012 strain. NS were subsequently tested for the presence of infectious virus in Vero E6 cells. Consistently, on 2 dpi, alpacas infected with the Jordan-1/2015 strain had remarkably higher viral titres than animals infected with EMC/2012. At both 2 and 3 dpi, only one out of three animals inoculated with the EMC/2012 strain shed infectious virus (Figure 1). Jordan-1/2015 strain, as opposed to the EMC/2012 strain, replicated better in the alpaca model, suggesting an increased transmission potential for clade B strains.

### MERS-CoV Jordan-1/2015 strain possesses an increased epithelial tropism along the respiratory tract compared to the EMC/2012 strain

**Figure 2.**
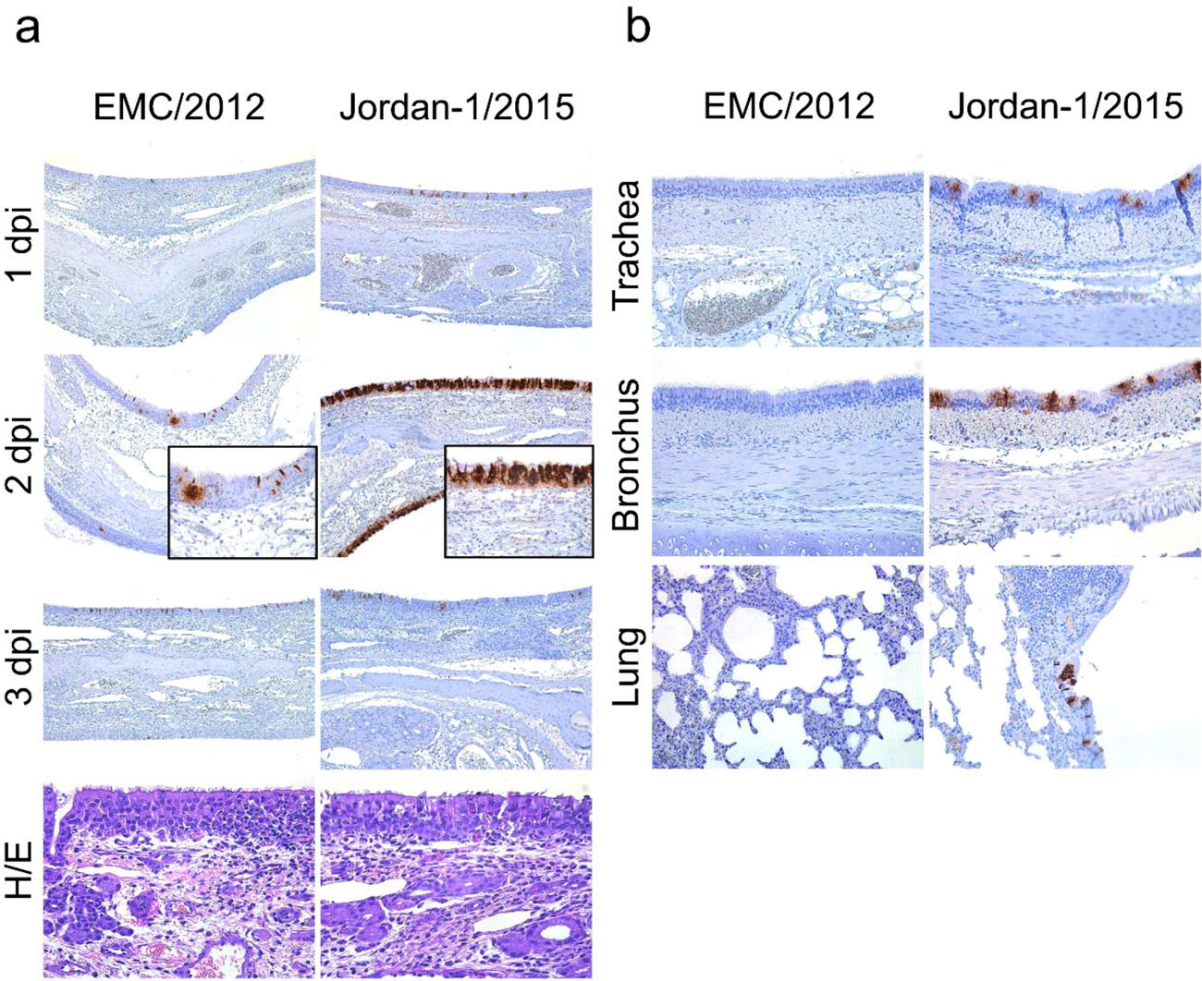
Histopathological changes and viral detection in respiratory tracts of alpacas inoculated with two MERS-CoV strains. Respiratory tissues (nasal turbinate, trachea, bronchus and lung) were harvested upon necropsy and immediately fixed in 10% neutral-buffered formalin. (a) Immunohistochemical (IHC) visualization of apical turbinate of alpacas infected with MERS-CoV EMC/2012 (left panel) and Jordan-1/2015 (right panel) euthanized and necropsied on 1 to 3 dpi. H/E staining of the apical nose collected on 2 dpi for both strains was displayed at the bottom. (b) IHC results of trachea, bronchus and lung of alpacas infected with MERS-CoV EMC/2012 (left panel) and Jordan-1/2015 (right panel) necropsied on 2 or 3 dpi. See Supplementary Table S2 for the detailed distribution of MERS-CoV antigen. Abbreviations: H/E, haematoxylin and eosin stain.

To gain further information on viral replication and tissue tropism we performed IHC for MERS-CoV detection along alpaca respiratory tracts infected with both strains. Viral antigen (N protein) was multifocally located in the nasal turbinates of alpacas inoculated with the MERS-CoV EMC/2012 strain (Figure 2(a)). On 1 and 3 dpi, MERS-CoV positive nasal epithelial cells were only detected in one out of three animals whereas on 2 dpi all three animals harboured viral antigen in their nose. By contrast, the virus was found in the nasal epithelium of all alpacas inoculated with the Jordan-1/2015 strain throughout the whole study (Supplementary Table S2). While a moderate number of pseudostratified columnar epithelial cells in the nose contained MERS-CoV Jordan-1/2015 antigen on 1 dpi, the number of positive epithelial cells became remarkably high on 2 dpi; such number steadily decreased in the alpacas necropsied on dpi 3 (Figure 2(a)).

MERS-CoV Jordan-1/2015 infected cells were unevenly detected within the tracheal and bronchial mucosa on 2 dpi by IHC (Figure 2(b)). Also, viral antigen was only scarcely found in bronchiolar epithelial cells of a single animal on 3 dpi (Figure 2(b) and Supplementary Table S2). By contrast, tissues from the trachea and the lower respiratory tract (LRT) were all negative by IHC in alpacas inoculated with the MERS-CoV EMC/2012 strain (Figure 2(b) and Supplementary Table S2).

Both MERS-CoV isolates led to similar degrees of histological lesions that were mainly limited to the nasal turbinate, being of multifocal distribution and mild. From 1 to 3 dpi, lesions in the nasal turbinates were characterized by mild rhinitis, segmental hyperplasia of the nasal epithelium, lymphocytic exocytosis and epithelial vacuolation. Additionally, small numbers of lymphocytes with fewer macrophages infiltrated the underlying submucosa (Figure 2(a)). No notable microscopic lesions were observed in the trachea and bronchus in response to both strains. Besides, lung lobes showed very mild multifocal perivascular and peribronchiolar infiltration by lymphocytes in few animals. Control animals euthanized on day 0 (AP19 to AP21) did not display any significant histological lesion in collected tissues and IHC resulted negative in all of them (Supplementary Table S2). Thus, the Jordan-1/2015 strain showed a better replication competence than did EMC/2012 along the alpaca respiratory tract, despite similar histopathological alterations provoked by both strains.

### MERS-CoV RNA loads in alpaca respiratory tracts

**Figure 3.**
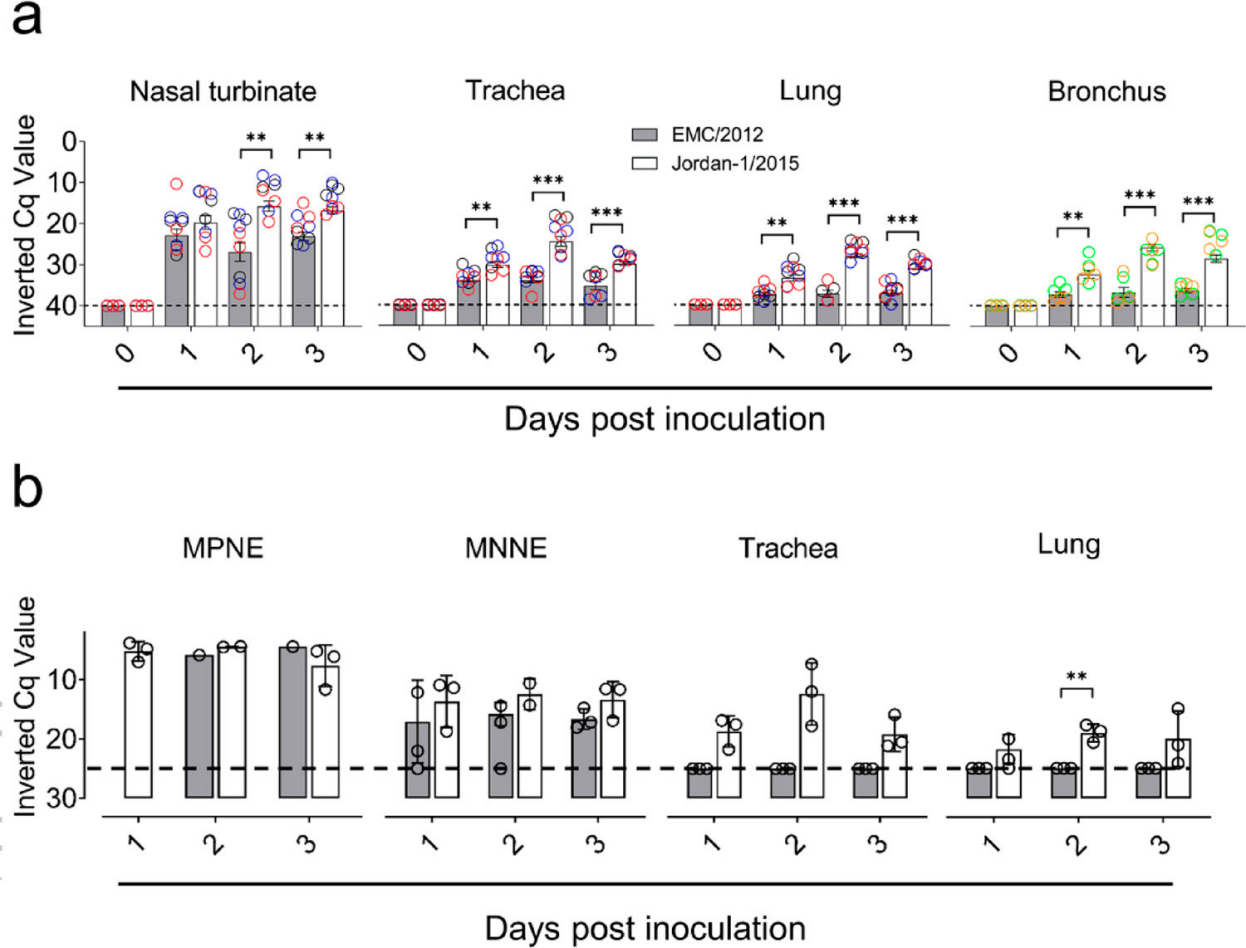
Viral loads in respiratory tissues of MERS-CoV EMC/2012 and Jordan-1/2015 infected alpacas. (a) Genomic viral loads from respiratory tissues of alpacas obtained at 0, 1, 2 and 3 dpi (1 to 3 dpi, n = 3 per day and per strain). Black circles: frontal turbinate, apical trachea and apical lung; blue circles: medial turbinate, medial trachea and medial lung; red circles: caudal turbinate, caudal trachea and caudal lung. Green and yellow circles represent values obtained from large and small bronchus, respectively. Genomic viral RNA loads were determined by the UpE RT-qPCR assay. Each bar represents the mean Cq value ± SEM of infected tissues from 3 animals euthanized on 0, 1, 2 and 3 dpi, respectively. Statistical significance was determined by Tukey's multiple comparisons test. *P < 0.05; **P < 0.01; ***P < 0.001; ****P < 0.0001. (b) Genomic viral RNA loads in MFPE samples. Micro-dissected MFPE-nasal epithelia were prepared based on an overlapping template section stained by IHC to localize the MERS-CoV N protein as described in Supplementary Figure 1. Nasal samples from AP14 sacrificed on 2 dpi could not be processed due to bad preservation. The trachea and lung were scrapped from MFPE tissue sections. RNA extracted from these samples were converted into cDNA and the MERS-CoV genomic RNA loads were determined by UpE primers in a microfluidic PCR assay (Fluidigm Biomark). Error bars indicate SEM when results were positive in more than one animal. Dashed lines depict the detection limit of the assays. Abbreviations: MPNE, MERS-CoV IHC positive nasal epithelia; MNNE, MERS-CoV IHC negative nasal epithelia; MFPE, methacarn-fixed paraffin embedded-tissues; LCM, laser capture microdissection. Cq, quantification cycle. Dashed lines depict the detection limit of the assays.

Upon infection with both strains, MERS-CoV genomic RNA was detected in non-fixed respiratory tissue specimens. The highest loads were found in nasal turbinates of Jordan-1/2015-inoculated animals at 2 and 3 dpi when compared to the EMC/2012 infected individuals. Viral loads were lower in the trachea, bronchus and lung for both strains, but animals that received the Jordan-1/2015, exhibited significantly higher rates of infection for the duration of the experiment (Figure 3(a)).

To ascertain active viral gene translation/replication within the whole respiratory tract, the presence of MERS-CoV subgenomic RNA in tissue macerates was assessed. Throughout the study, M mRNA loads were significantly higher in nasal turbinates of the Jordan-1/2015 inoculated alpacas. In contrast to group 2 animals, subgenomic RNA was not detected in the trachea, bronchus and lung of alpacas inoculated with the EMC/2012 strain (Supplementary Figure 2(a)). These data corroborated results obtained by IHC and confirmed that the Jordan-1/2015 strain replicated better than the EMC/2012 strain in all respiratory tissues from alpacas, in particular during the peak of infection (2 dpi).

Loads of MERS-CoV genomic UpE and subgenomic M mRNA were also evaluated by a microfluidic RT-qPCR assay on individual MFPE respiratory tissue sections or micro-dissected mucosa that were used also for cytokine mRNA quantification. Since the nasal mucosa is the major driver of innate immune responses in MERS-CoV infected alpacas [29], LCM was used to isolate MERS-CoV infected/non-infected nasal epithelial areas based on IHC examination. Also, MFPE trachea and lung sections were directly scrapped from the slide. Due to a lower infectivity of the EMC/2012 strain, extended nasal epithelial areas labelled by IHC could only be harvested from two animals (AP4 on 2 dpi and AP9 on 3 dpi). Nasal specimens from animal AP14, infected with the Jordan-1/2015 strain, could not be examined due to bad preservation. All other nasal epithelia retrieved from animals inoculated by the Jordan-1/2015 strain could be processed as expected. As shown in Figure 3(b) and Supplementary Figure 2(b), viral genomic and subgenomic RNA loads found in MERS-CoV IHC positive nasal epithelial (MPNE) areas were higher than those displayed by MERS-CoV IHC negative nasal epithelial (MNNE) areas. In addition, MPNE micro-dissected areas of both strains showed comparable viral RNA loads, while the Jordan-1/2015 strain had a slightly better replication in MNNE areas than that of EMC/2012. Similar to results obtained from tissue homogenates (Figure 3(a)), genomic and subgenomic viral RNA was absent in trachea and lung sections of alpacas inoculated with the EMC/2012 strain. In contrast, genomic and subgenomic RNA loads were observed in trachea and lung section of animals infected with the Jordan-1/2015 strain (Figure 3(b) and Supplementary Figure 2(b)).

### Expression of innate immune genes in the nasal epithelia of alpacas infected with the EMC/2012 or Jordan-1/2015 strains

**Figure 4.**
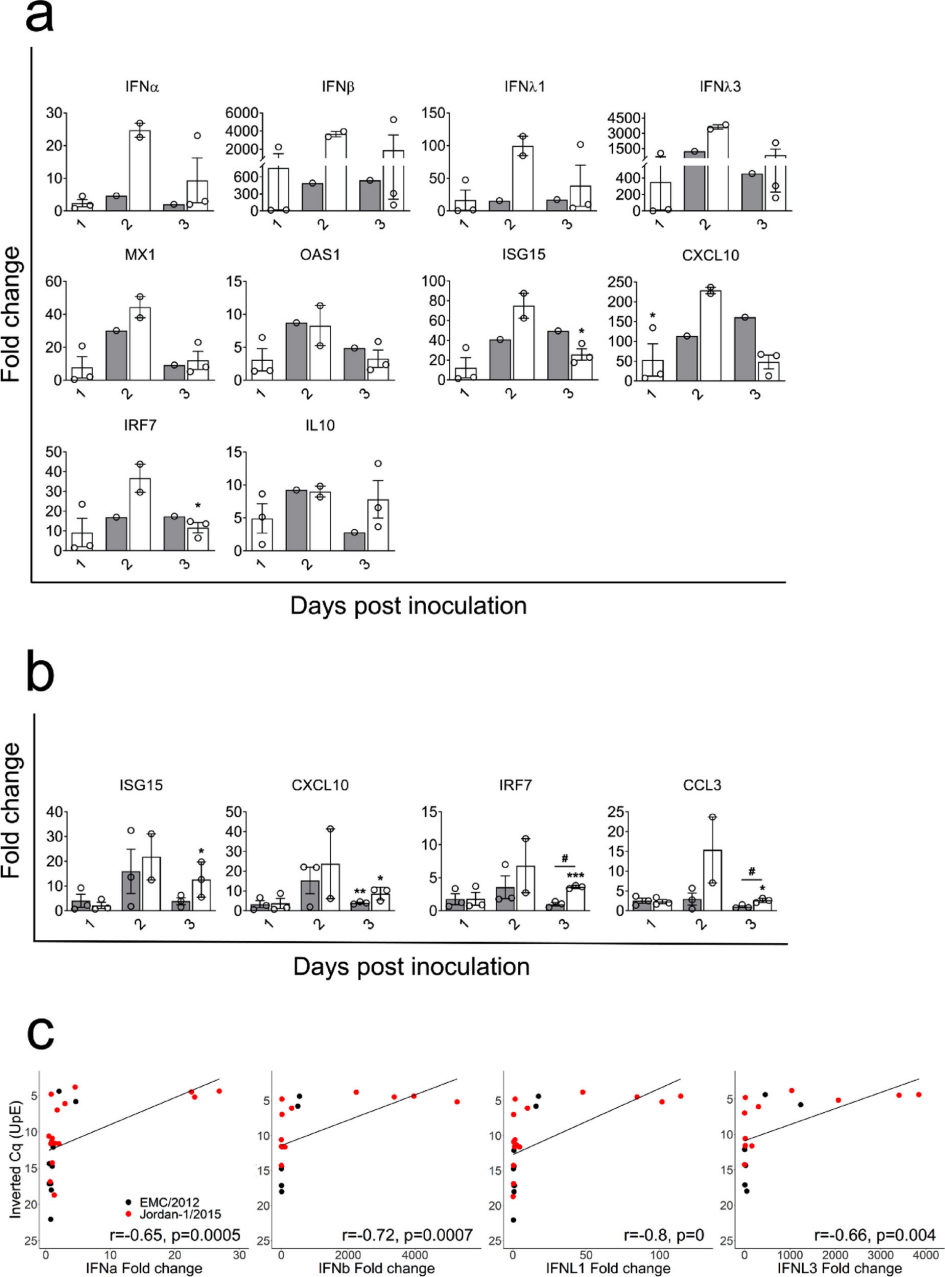
Kinetics of innate immune response genes and correlation coefficients between MERS-CoV genomic RNA load and IFN induction in alpaca nasal epithelia. The nasal epithelia of each alpaca (AP1 to AP21, except for AP14) was micro-dissected and MPNE and MNNE areas were selected and isolated for RNA extraction and conversion to cDNA. The Fluidigm Biomark microfluidic assay was used to amplify and quantify transcripts of innate immune genes at different dpi (1 to 3 dpi). *HPRT1*, *GAPDH* and *UBC* genes were used as normalizer genes, and values obtained from the infected animals were compared to those obtained from non-infected alpacas. Average Fc of genes in MERS-CoV positive nasal epithelia (a) and negative nasal epithelia (b) of EMC/2012 (gray bars) and Jordan-1/2015 (white bars) were shown. Data were displayed as means of ± SEM. Statistical significance was determined by Student’s *t*-test. *P < 0.05; **P < 0.01; ***P < 0.001 (*n* = 3) compared with non-infected alpacas; #*P* < 0.05; ##*P* < 0.01; ###*P* < 0.001 (*n* = 3) compared between groups on different dpi. (c) Cq values of MERS-CoV genomic RNA in nasal epithelia were plotted against the relative expression levels of IFNα, IFNβ, IFNλ1 and IFNλ3. Black and red spheres represent samples obtained from EMC/2012 and Jordan-1/2015 strains infected groups, respectively. Correlation coefficients were established using Spearman’s correlation test.

To compare antiviral and inflammatory pathways induced by both strains, relative mRNA expression levels for 37 innate immune response genes [29] were monitored in the upper respiratory tract (URT) and LRT tissues upon infection. All genes were detected at basal levels in micro-dissected samples of control non-infected animals. In MPNE areas, type I and III IFNs were induced as early as 1 dpi in alpacas from group 2. However, MPNE areas could not be collected on 1 dpi due to the paucity of MERS-CoV EMC/2012 infected epithelial cells in group 1 animals. On 2 and 3 dpi, IFNs were upregulated in the MPNE areas infected by both strains. Of note, the Jordan-1/2015 strain induced much higher relative levels of type I and III IFNs (Figure 4(a) and Supplementary Figure 3(a)). Genes coding for pattern recognition receptors (PRRs) including RIG1 and MDA5, antiviral ISGs (OAS1, CXCL10, MX1 and ISG15), chemokines (CCL2 and CCL3), the transcriptional factors IRF7 and the NLRP3 inflammasome component CASP1 were highly upregulated on 2 dpi and levels of expression decreased on 3 dpi. The same kinetic of mRNA transcription but with a lower induction was observed for the pro-inflammatory cytokines (IL1β, IL6, IL8, IL15, TNFα and CXCL1) and the anti-inflammatory gene IL10 in MPNE areas infected by both strains. Nonetheless, levels of expression of these cytokines could drastically vary between animals (Figure 4(a), Supplementary Figure 3(a, b) and Supplementary Data S1). Additionally, transcription levels of genes coding for the NLRP3 inflammasome components NLRP3 and PYCARD, downstream signalling adaptors (NFκBIA and CARD9), enzymes (CASP10, AZI2, PACT and TBK1) and the IFNLR1 receptor fluctuated around basal values independently of the animals, days of sample collection and the strain used for infection (Supplementary Figure 3(a) and Supplementary Data S1).

Type I and III IFNs were not detected or marginally upregulated in MNNE areas from alpacas inoculated by both strains. On 2 dpi, corresponding to the peak of infection, expression of ISGs, RLRs, STAT1, IRF7, IL8, CCL2, CCL3 and CASP1 were moderate to highly induced in MNNE areas from animals infected by both strains while most of the remaining genes were unaltered or slightly down-regulated upon infection (Figure 4(b), Supplementary Figure 3(a, c) and Supplementary Data S1). Again, high variations between animals within groups were noticed.

In a previous study [29], we found that transcriptional levels of IFNs were positively correlated with increased viral loads in the nasal mucosa of alpacas inoculated with the MERS-CoV Qatar15/2015 strain. To confirm this relation for the Jordan-1/2015 and the EMC/2012 strains, mRNA levels of IFNs, expressed in Fc, were plotted against Cq values found in the MERS-CoV UpE microfluidic PCR assay for both MPNE and MNNE areas. As shown in Figure 4(c), genomic RNA in micro-dissected tissues were strongly or moderately correlated to the inductions of IFNβ (*r* = 0.72, *P* = 0.0007) and IFNλ1 (*r* = 0.8, *P* < 0.0001), or to IFNα (*r* = 0.65, *P* = 0.0005) and IFNλ3 (*r* = 0.66, *P* = 0.004), respectively. Thus, our previous finding showing that the expression of IFNs depended on levels of viral replication was confirmed and extended to the MERS-CoV EMC/2012 clade A strain.

To further explore whether the Jordan-1/2015 strain induces higher IFN responses, IFN relative quantification values from nasal epithelia of alpacas infected with Clade B MERS-CoV Qatar15/2015 [29] and Jordan-1/2015 strains were normalized against the mean of six non-infected control animals, including three animals from a previous study [29]. No significant variability was observed on IFN levels among the six control animals, which allowed normalization procedures to be considered for the data sets obtained with both strains (Supplementary Table S3). Interestingly, no IFNs were induced until viral loads reached a threshold of Cq = 6.46 (Supplementary Figure 4(a)). Thus, MPNE samples with Cq < 6.46 were chosen to compare their IFN levels. As indicated in Supplementary Figure 4(b), the Jordan-1/2015 strain induced significantly higher relative levels of IFNλ1 than did the Qatar15/2015 strain despite similar viral loads. Moreover, same differences were observed between both strains for levels of expression of IFNα, β and λ3, although the results were not statistically significant. Considering that both viral strains mainly differ by the ORF4a sequence and that the Qatar15/2015 strain does not induce IFN mRNAs at 1 dpi, this accessory protein may inhibit temporally earlier IFN transcription but may have only a moderate antagonistic role in IFN responses during the course of infection

### Innate immune response genes follow the same expression kinetics in the trachea and lung upon infection with the EMC/2012 and Jordan-1/2015 strains

**Figure 5.**
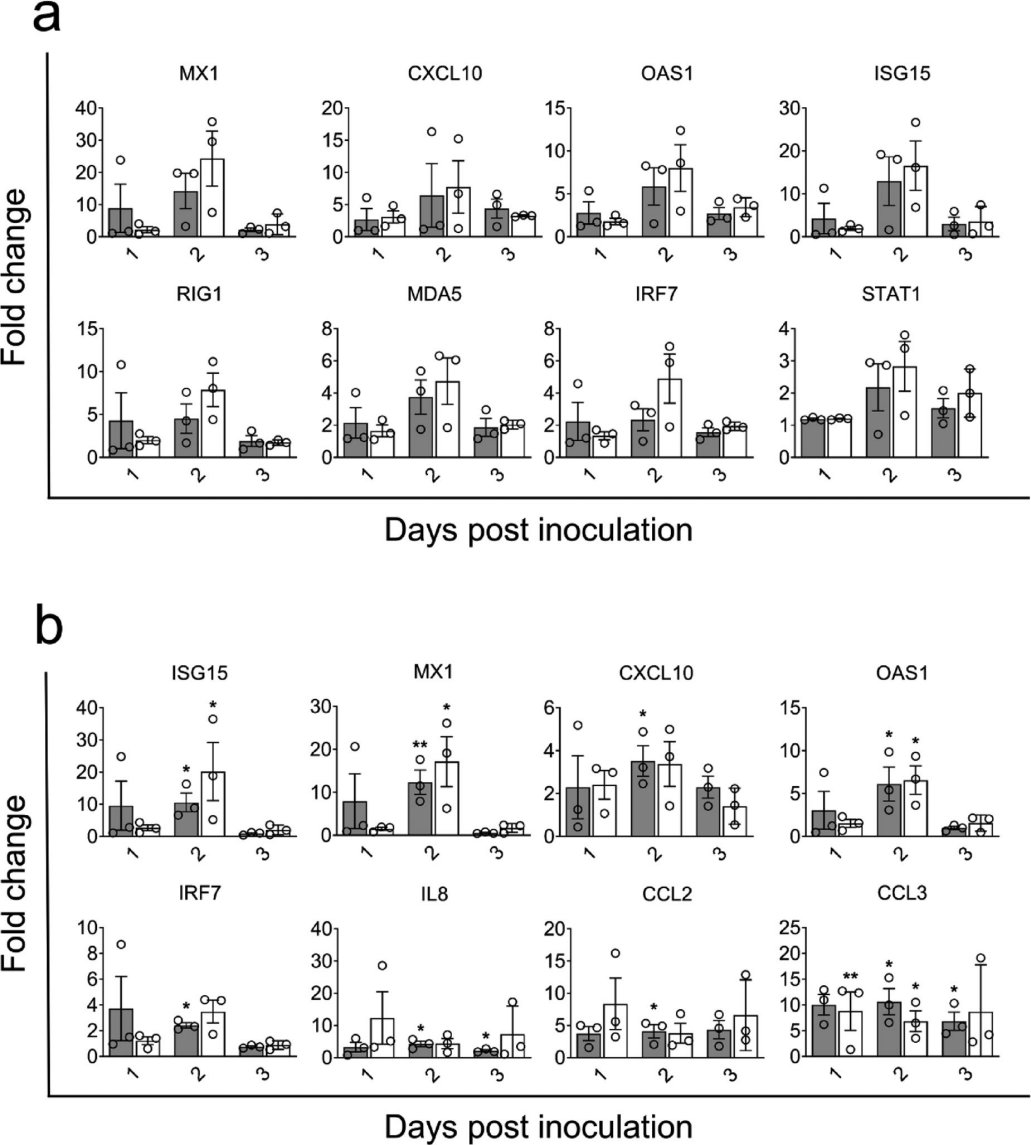
Kinetics of innate immune response genes in the trachea and lung of alpacas upon infection with MERS-CoV EMC/2012 and Jordan-1/2015 strains. Trachea and lung samples were obtained by scraping MFPE sections from control and infected alpacas (AP1 to AP21). After RNA extraction and conversion to cDNA, a Fluidigm Biomark microfluidic assay was used to amplify and quantify transcripts of innate immune genes at different dpi (1 to 3 dpi). *HPRT1, GAPDH* and *UBC* genes were used as normalizers, and values obtained from the infected animals were compared to those obtained from noninfected alpacas. Average fold change of highly induced genes in the trachea (a) and the lung (b) of alpacas infected with EMC/2012 (gray bars) and Jordan-1/2015 (white bars) were shown. Data were displayed as means of ±SEM. Statistical significance was determined by Student’s *t*-test. *P < 0.05; **P < 0.01; ***P < 0.001 (*n* = 3) compared with non-infected alpacas.

Expression of innate immune response genes was further assessed along the URT and LRT. Except for IFNλ3, which was not expressed, all other genes were detected at basal levels in the trachea and lung of control non-infected animals. Transcription of all IFNs was not induced in the trachea and lungs of alpacas upon infection with any of the strains (Supplementary Figure 5(a, b) and Supplementary Data S1). In the trachea, expression of PRRs, ISGs, IRF7 and STAT1 were moderately up regulated in both groups during the course of infection (Figure 5(a) and Supplementary Figure 5(a)). Compared to non-infected controls, there were few alterations of mRNA transcription in the remaining genes tested during the infection period (Supplementary Figure 5(a) and Supplementary Data S1).

In the lung, the EMC/2012 and Jordan-1/2015 strains induced the same pattern of gene transcription. For both strains, ISGs, RLRs (RIG1 and MDA5), IRF7, IL8, CXCL1, CCL2, CCL3 and CASP10 were slightly to moderately upregulated from 1 dpi, reaching a peak on 2 dpi to further decay on 3 dpi (Figure 5(b) and Supplementary Figure 5(b)). However, as for the trachea, animals from group 2 tended to show higher induction of the above-mentioned cytokines. The rest of the genes fluctuated around basal levels independently of the animals and days of sample collection (Supplementary Figure 5(b) and Supplementary Data S1).

### Protein alignment of selected clade A and B MERS-CoV strains and protein structure mapping

**Figure 6.**
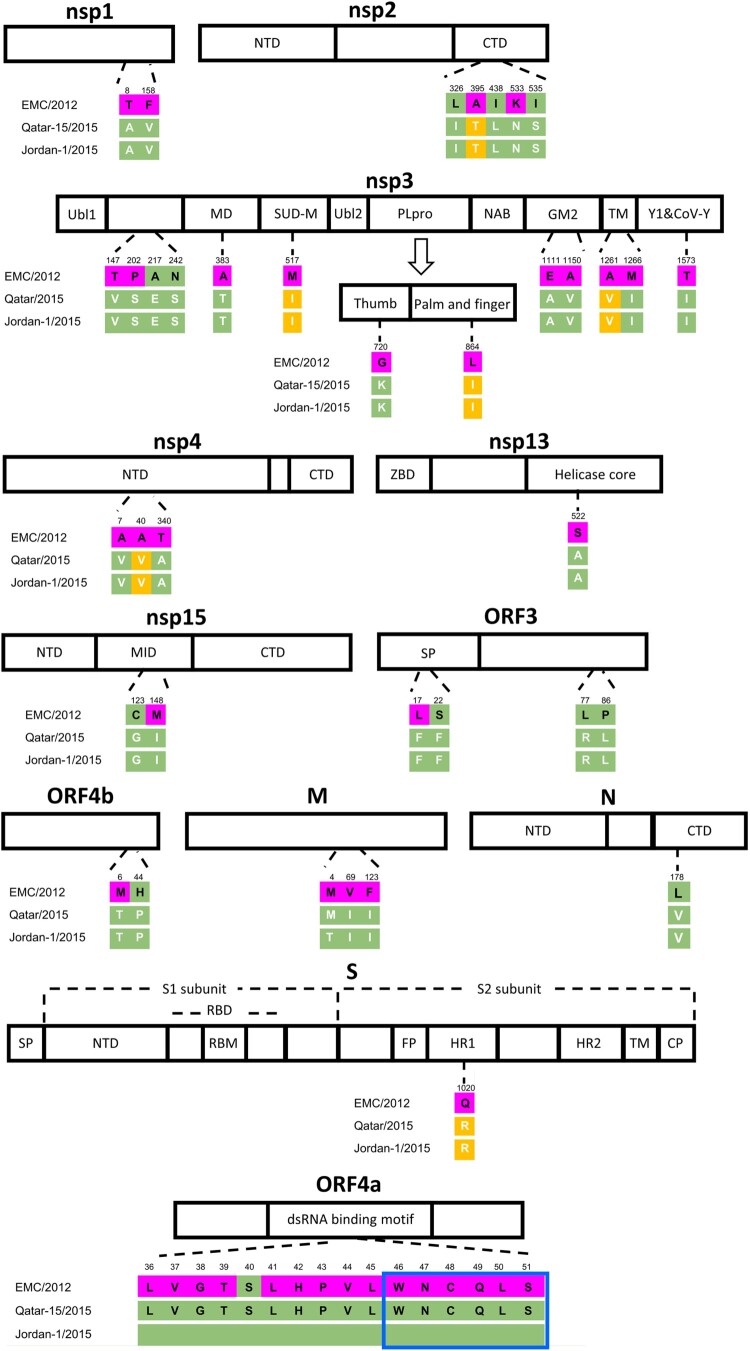
Schematic diagram of divergent amino acids found in proteins of MERS-CoV clade A and B strains. Full aa sequences of twenty-one MERS-CoV strains were aligned with the UniProt alignment tool. Differences in aa residues affecting each protein are indicated by single-letter aa codes with positions indicated at the top of the diagram. The black and white letters indicate the aa of the prototype strain EMC/2012 and the clade B strains (Qatar15/2015 and Jordan-1/2015), respectively. The conserved aa sequences within clade A and B strains were highlighted in purple and yellow, respectively. The predicted β-sheet belonging to the classical double-stranded RNA binding αβββα fold of ORF4a is blue boxed. Abbreviations: nsp, non-structural protein; NTD, N terminal domain; CTD, C terminal domain; Ubl, ubiquitin-like domain; MD, Macro domain; SUD-M, middle region of SARS-CoV unique domain; PLpro, papain-like protease; NAB, nucleic-acid binding domain; GM2, betacoronavirus-specific marker; TM, transmembrane domain; ZBD, Zinc binding domain; MID, middle domain; SP, signal peptidase; RBD, receptor-binding domain; RBM, receptor-binding motif; FP, fusion peptide; HR, heptad repeat; CP, cytoplasmic domain.

**Figure 7.**
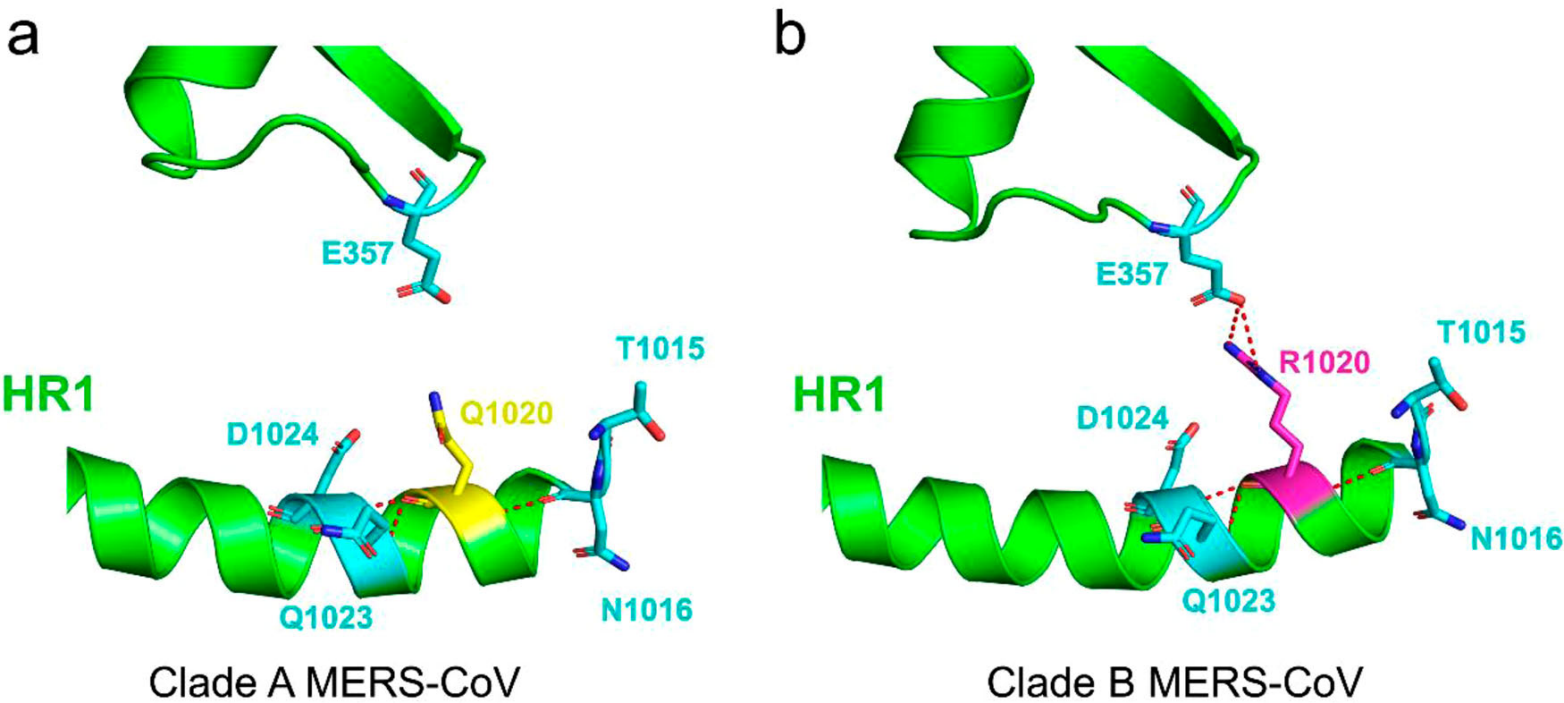
Comparative structural view of the S protein HR1 region in clade A and B MERS-CoV strains. Details of inter- and intra-helical interactions for residue 1020 of the MERS-CoV spike protein (PDB ID: 5W9H) in clade A (a) and B (b) strains. Q1020 (yellow), R1020 (magenta) and aa residues proximal to 1020 (light blue) are shown as sticks; Polar contacts with residue 1020 are shown as red dot lines. The figures were produced using PyMOL.

To explore whether the increased replication competence of the Jordan-1/2015 strain could be related to clade B-specific mutations, all protein coding genes of two MERS-CoV clade A and nineteen clade B strains, encompassing lineages 1 to 7 (Table 1), were randomly selected and compared using the UniProt alignment tool. All selected MERS-CoV clade B strains share 5 specific aa mutations in the replicase genes of ORF1ab (Figure 6). Of note, in nsp3, a L864I substitution occurred at the palm and fingers subdomains of PLpro (Figure 6) that cleaves the viral replicase polyproteins at three sites releasing viral nsp1, nsp2, and nsp3. Comparative protein structure analysis using PyMOL indicated that the L864I mutation is distant from the PLpro active site and thus might not affect polar contacts with nearby residues (Supplementary Figure 6). Further, all MERS-CoV clade B strains show a Q1020R substitution in the heptad repeat (HR) 1 of the S protein (Figure 6). Interestingly, this mutation allows the formation of hydrogen bonds with the residue E357, located between the N terminal domain (NTD) and the receptor-binding domain (RBD), and may affect the fusion mechanism (Figure 7). The remaining aa sequences of clade A strains were highly conserved at nsp2, nsp3 and nsp4 where all 4 clade B-specific mutations occurred. All in all, compared with the EMC/2012 strain, the two clade B viruses used so far to infect experimentally alpacas (Qatar15/2015 and Jordan-1/2015) share 36 specific aa mutations, mostly found in the ORF1ab gene and to a lesser extent, in the ORF3, the membrane protein (M), the ORF4a, the nucleocapsid (N) and the spike (S) gene products. The Jordan-1/2015 harboured a unique 16-aa deletion in ORF4a, which is not found in all clade A and B strains analysed. The deleted region contains a predicted β-sheet belonging to the classical double-stranded RNA binding αβββα fold of this protein (Figure 6, boxed in blue). Overall, the genomic and the structural protein prediction analysis revealed that clade B viruses possess several mutations that could affect the viral fusion with plasmatic membranes, viral replication and host antiviral responses such as the IFN signalling cascade.

## Discussion

In the Arabian Peninsula, all contemporary zoonotic MERS-CoV strains belong to clade B. Hence, the evolution of clade B viruses throughout the epidemic must be an advantage over early epidemic clade A strains in the reservoir host. RNA viruses are prone to undergo natural selection to create advantageous variants due to a combination of high mutation rates and large population sizes [35,36]. Indeed, the dromedary population is large in the Middle East and with a very high seroprevalence of MERS-CoV [37]. Here, our study provides a rational explanation on why MERS-CoV B strains substituted clade A strains in the field. We compared the viral replication competence, tissue tropism and induced innate host immune responses of the prototype MERS-CoV strain EMC/2012 (clade A) to the Jordan-1/2015 strain (clade B), all isolated from humans, in a camelid host model. None of the inoculated alpacas had clinical signs at any time, and no macroscopic lesions were observed upon infection with both strains. However, alpacas challenged with the Jordan-1/2015 strain had higher titres of infectious virus in their nasal cavity than did the EMC/2012 strain, suggesting a higher transmission capability of clade B strains. Indeed, an increased replication capacity of the Jordan-1/2015 strain was observed in the nasal epithelia of infected alpacas. Moreover, compared to the EMC/2012 strain, the Jordan-1/2015 strain exhibited a broader tissue tropism, showing productive infection in tracheal, bronchial and bronchiolar epithelial cells. In fact, the clade B Qatar15/2015 strain, which was used in a previous study, had comparable replication competences in the nasal mucosa as observed with the Jordan-1/2015 strain [29]. Thus, clade B viral strains are displaying a better replicative fitness in camelids than the clade A EMC/2012 strain.

At the first 24 h of infection, type I or III IFNs were moderate to highly induced in the nasal mucosa of alpacas in response to the Jordan-1/2015 strain but not to the EMC/2012 strain (Supplementary Data S1). Our previous results with the Qatar15/2015 strain showed that only IFNβ started to be transcribed at very low levels at 1 dpi [29]. Thus, such an early IFN response could be due to the partial aa deletion in the ORF4a protein affecting the Jordan-1/2015 strain but does not interfere with the early replication capability of this strain in the nasal mucosa. Indeed, antiviral ISGs, such as MX1, OAS1 or ISG15, were maximally expressed at 2 dpi coinciding with a dramatic decay of viral loads in nasal tissues at 3 dpi. Of note, the deletion found in the Jordan-1/2015 ORF4a contains a predicted β-sheet that is suspected to affect the binding of dsRNA [31]. By contrast, the EMC/2012 and the Qatar15/2015 strains harbour intact ORF4a sequences. In addition, the peak of MERS-CoV replication and maximal IFN transcription coincided in all three strains on 2 dpi. We confirmed that enhanced IFN expression levels correlated with higher viral replication in nasal tissues; but, despite similar viral loads found in MPNE areas, the Jordan-1/2015 strain had only a moderate impact on IFN mRNA transcription when compared to the Qatar15/2015 strain. Together, our data suggest that the IFN antagonistic role of the ORF4a protein is exerted *in vivo* only during the first hours following infection. However, more experiments are needed to definitely ascertain the role of ORF4a *in vivo*.

Despite an enhanced replication competence and early IFN induction in the nasal mucosa, the Jordan-1/2015 strain led to similar moderate pro-inflammatory responses as those provoked by the EMC/2012 strain along alpaca respiratory tracts. As observed with the Qatar15/2015 strain [29], and contrary to ISGs, IFNs were not induced at any time of the infection in trachea or lungs, supporting the hypothesis that IFNs produced in the nasal mucosa can act in an endocrine manner. Additionally, the same histopathological alterations were noted in nasal turbinates of alpacas infected by the strains used in the present study and were equal to those caused by the Qatar15/2015 strain [29]. Differences between the Qatar15/2015 and the Jordan-1/2015 strains resided in higher numbers of IHC MERS-CoV positive epithelial cells in the LRT and larger infiltration of leukocytes in the lungs of alpacas undergoing an infection with the Qatar15/2015 strain. This might be due to an earlier IFN response provoked by the Jordan-1/2015 strain. By contrast, the EMC/2012 strains did not replicate within the alpaca lungs, suggesting that the Qatar15/2015 strain might be the most virulent MERS-CoV experimentally tested so far in camelids. Indeed, as opposed to results obtained in the present study, upregulation of pro-inflammatory genes such as NLRP3, TNFa and IL15 was observed in lungs of Qatar15/2015 infected animals [29]. By contrast, in lungs of h-DPP4 transduced mice infected with the clade B strain, ChinaGD01, only IRF3, IRF5 and IL15 were significantly upregulated in relation to the EMC/2012 strain. More severe inflammation was reported in the lung of mice infected with the ChinaGD01 strain but no differences in IFN transcription levels were observed between the two strains [19].

Based on the comparison of coding sequences between all clade A and representative clade B strains, it appears that multiple punctual aa mutations are mostly distributed along the replicase gene. One of the most striking changes differentiating clade A and B strains is the L864I substitution at the palm and fingers subdomain of PLpro of nsp3 but, unless involved in substrate binding, it might not alter the cleavage of viral polyproteins during replication. Likewise, the other four clade B-specific mutations affecting nsp2 (A395T), nsp3 (M517I and A1261V) and nsp4 (A40 V) may have marginal effect on the viral fitness since these sites do not belong to any of residues involving MERS-CoV replication machinery. Remarkably, a key clade B-specific mutation (Q1020R) happened in HR1, essential for the fusion mechanism of the S protein allowing passage of the virus across the endosomal membrane [38]. In fact, Arginine at position 1020 of the S protein was believed to be the hallmark of clade B strains [39] and was positively selected during virus evolution [18]. Protein structure analysis revealed that R1020, instead of Q1020, could interact with E357 by hydrogen bonds providing a protonated residue and a potential protease cleavage site that may affect the S protein membrane fusion process. Forni *et al.* reported that Q1020R replacement reduces the stability of a six-helix bundle formation in the post-fusion conformation [40]. A similar level of destabilization was also observed for a nearby mutation (T1015N), which increases MERS-CoV infection efficiency under *in vitro* condition [40,41]. Indeed, whether the interaction between R1020 and E357 is responsible for the destabilization process and influences fusion intermediate or pre-fusion state of S protein requires further experimental proof. Our results taken together, with data obtained by others [40,41], point out the possible role of the Q1020R substitution for an enhanced replicative capability of MERS-CoV clade B strains. Overall, we showed experimental evidence that early epidemic clade A strains could be outcompeted by contemporary viruses due to a higher replication fitness in camelid populations. Nonetheless, none of the strains tested so far in camelids caused disease in these species, but rather elicited an effective innate immune response based on early recruitment of antiviral mechanisms and a controlled inflammation. Further reverse genetic studies are needed to unravel the consequences of mutations differentiating MERS-CoV clades and influencing increased replication capability as their interaction with host components of innate immune pathways.

## Supplementary Material

Supplemental Material

Supplemental Material

## References

[1] Memish ZA, Perlman S, Van Kerkhove MD, et al. Middle East respiratory syndrome. Lancet. 2020;395:1063–1077. 32145185 10.1016/S0140-6736(19)33221-0 PMC7155742

[2] Ministry of Health – Kingdom of Saudi Arabia. https://www.moh.gov.sa/en/CCC/events/national/Pages/2020.aspx (accessed 23 Jun2021).

[3] WHO. Middle East respiratory syndrome coronavirus (MERS-CoV). https://www.who.int/emergencies/mers-cov/en/ (accessed 18 Jun2021).

[4] Kim KH, Tandi TE, Choi JW, et al. Middle East respiratory syndrome coronavirus (MERS-CoV) outbreak in South Korea, 2015: epidemiology, characteristics and public health implications. J Hosp Infect. 2017;95:207–213. 28153558 10.1016/j.jhin.2016.10.008 PMC7114867

[5] Oh M, Park WB, Park S-W, et al. Middle East respiratory syndrome: what we learned from the 2015 outbreak in the Republic of Korea. Korean J Intern Med. 2018;33:233–246. 29506344 10.3904/kjim.2018.031 PMC5840604

[6] Adney DR, Bielefeldt-Ohmann H, Hartwig AE, et al. Infection, replication, and transmission of Middle East respiratory syndrome coronavirus in alpacas. Emerg Infect Dis. 2016;22:1031–1037. 27070385 10.3201/eid2206.160192 PMC4880070

[7] Crameri G, Durr PA, Klein R, et al. Experimental infection and response to rechallenge of alpacas with Middle East respiratory syndrome coronavirus. Emerg Infect Dis. 2016;22:1071–1074. 27070733 10.3201/eid2206.160007 PMC4880109

[8] Reusken CBEM, Schilp C, Raj VS, et al. MERS-CoV infection of alpaca in a region where MERS-CoV is endemic. Emerg Infect Dis. 2016;22:1129–1131. 27070501 10.3201/eid2206.152113 PMC4880085

[9] David D, Rotenberg D, Khinich E, et al. Middle East respiratory syndrome coronavirus specific antibodies in naturally exposed Israeli llamas, alpacas and camels. One Heal. 2018;5:65–68. 10.1016/j.onehlt.2018.05.002 PMC6000904 29911167

[10] Vergara-Alert J, van den Brand JMA, Widagdo W, et al. Livestock susceptibility to infection with Middle East respiratory syndrome coronavirus. Emerg Infect Dis. 2017;23:232–240. 27901465 10.3201/eid2302.161239 PMC5324816

[11] Rodon J, Okba NMA, Te N, et al. Blocking transmission of Middle East respiratory syndrome coronavirus (MERS-CoV) in llamas by vaccination with a recombinant spike protein. Emerg Microbes Infect. 2019;8:1593–1603. 31711379 10.1080/22221751.2019.1685912 PMC6853226

[12] Azhar EI, El-Kafrawy SA, Farraj SA, et al. Evidence for camel-to-Human transmission of MERS coronavirus. N Engl J Med. 2014;370:2499–2505. 24896817 10.1056/NEJMoa1401505

[13] Killerby ME, Biggs HM, Midgley CM, et al. Middle East respiratory syndrome coronavirus transmission. Emerg Infect Dis. 2020;26:191–198. 31961300 10.3201/eid2602.190697 PMC6986839

[14] Chu DKW, Hui KPY, Perera RAPM, et al. MERS coronaviruses from camels in Africa exhibit region-dependent genetic diversity. Proc Natl Acad Sci. 2018;115:3144–3149. 29507189 10.1073/pnas.1718769115 PMC5866576

[15] Kiambi S, Corman VM, Sitawa R, et al. Detection of distinct MERS-coronavirus strains in dromedary camels from Kenya, 2017. Emerg Microbes Infect. 2018;7:1–4. 30482895 10.1038/s41426-018-0193-z PMC6258726

[16] Sabir JSM, Lam TT-Y, Ahmed MMM, et al. Co-circulation of three camel coronavirus species and recombination of MERS-CoVs in Saudi Arabia. Science. 2016;351:81–84. 26678874 10.1126/science.aac8608

[17] El-Kafrawy SA, Corman VM, Tolah AM, et al. Enzootic patterns of Middle East respiratory syndrome coronavirus in imported African and local Arabian dromedary camels: a prospective genomic study. Lancet Planet Heal. 2019;3:e521–e528. 10.1016/S2542-5196(19)30243-8 PMC6926486 31843456

[18] Naeem A, Hamed ME, Alghoribi MF, et al. Molecular evolution and structural mapping of N-terminal domain in spike gene of Middle East respiratory syndrome coronavirus (MERS-CoV). Viruses. 2020;12:502. 10.3390/v12050502 PMC7290774 32370153

[19] Wang Y, Sun J, Li X, et al. Increased pathogenicity and virulence of Middle East respiratory syndrome coronavirus clade B in vitro and in vivo. J Virol. 2020;94:e00861-20. 32434886 10.1128/JVI.00861-20 PMC7375386

[20] Chan RWY, Chu DKW, Poon LLM, et al. Tropism and replication of Middle East respiratory syndrome coronavirus from dromedary camels in the human respiratory tract: An in-vitro and ex-vivo study. Lancet Respir Med. 2014;2:813–822. 25174549 10.1016/S2213-2600(14)70158-4 PMC7164818

[21] Yang Y, Zhang L, Geng H, et al. The structural and accessory proteins M, ORF 4a, ORF 4b, and ORF 5 of Middle East respiratory syndrome coronavirus (MERS-CoV) are potent interferon antagonists. Protein Cell. 2013;4:951–961. 24318862 10.1007/s13238-013-3096-8 PMC4875403

[22] Matthews KL, Coleman CM, van der Meer Y, et al. The ORF4b-encoded accessory proteins of Middle East respiratory syndrome coronavirus and two related bat coronaviruses localize to the nucleus and inhibit innate immune signalling. J Gen Virol. 2014;95:874–882. 24443473 10.1099/vir.0.062059-0 PMC3973478

[23] Yang Y, Ye F, Zhu N, et al. Middle East respiratory syndrome coronavirus ORF4b protein inhibits type I interferon production through both cytoplasmic and nuclear targets. Sci Rep. 2015;5:17554. 26631542 10.1038/srep17554 PMC4668369

[24] Mielech AM, Kilianski A, Baez-Santos YM, et al. MERS-CoV papain-like protease has deISGylating and deubiquitinating activities. Virology. 2014;450–451:64–70. 10.1016/j.virol.2013.11.040 PMC3957432 24503068

[25] Lokugamage KG, Narayanan K, Nakagawa K, et al. Middle East respiratory syndrome coronavirus nsp1 inhibits host gene expression by selectively targeting mRNAs transcribed in the nucleus while sparing mRNAs of cytoplasmic origin. J Virol. 2015;89:10970–10981. 26311885 10.1128/JVI.01352-15 PMC4621111

[26] Niemeyer D, Zillinger T, Muth D, et al. Middle East respiratory syndrome coronavirus accessory protein 4a is a type I interferon antagonist. J Virol. 2013;87:12489–12495. 24027320 10.1128/JVI.01845-13 PMC3807936

[27] Comar CE, Goldstein SA, Li Y, et al. Antagonism of dsRNA-induced innate immune pathways by NS4a and NS4b accessory proteins during MERS coronavirus infection. MBio. 2019;10:e00319-19. 30914508 10.1128/mBio.00319-19 PMC6437052

[28] Siu K-L, Yeung ML, Kok K-H, et al. Middle East respiratory syndrome coronavirus 4a protein Is a double-stranded RNA-binding protein that suppresses PACT-induced activation of RIG-I and MDA5 in the innate antiviral response. J Virol. 2014;88:4866–4876. 24522921 10.1128/JVI.03649-13 PMC3993821

[29] Te N, Rodon J, Ballester M, et al. Type I and III IFNs produced by the nasal epithelia and dimmed inflammation are features of alpacas resolving MERS-CoV infection. PLOS Pathog. 2021;17:e1009229. 34029358 10.1371/journal.ppat.1009229 PMC8195365

[30] van Boheemen S, de Graaf M, Lauber C, et al. Genomic characterization of a newly discovered coronavirus associated with acute respiratory distress syndrome in humans. MBio. 2012;3:e00473-12. 23170002 10.1128/mBio.00473-12 PMC3509437

[31] Lamers MM, Raj S V., Shafei M, et al. Deletion variants of Middle East respiratory syndrome coronavirus from humans, Jordan, 2015. Emerg Infect Dis. 2016;22:716–719. 26981770 10.3201/eid2204.152065 PMC4806954

[32] Corman VM, Eckerle I, Bleicker T, et al. Detection of a novel human coronavirus by real-time reverse-transcription polymerase chain reaction. Eurosurveillance. 2012;17:20285. 23041020 10.2807/ese.17.39.20285-en

[33] Coleman CM, Frieman MB. Growth and quantification of MERS-CoV infection. Curr Protoc Microbiol. 2015;37:1521–1529. doi:10.1002/9780471729259.mc15e02s37. PMC4735735 26344219

[34] Te N, Vergara-Alert J, Lehmbecker A, et al. Co-localization of Middle East respiratory syndrome coronavirus (MERS-CoV) and dipeptidyl peptidase-4 in the respiratory tract and lymphoid tissues of pigs and llamas. Transbound Emerg Dis. 2019;66:831–841. 30520548 10.1111/tbed.13092 PMC7027813

[35] Holmes EC. The evolution and emergence of RNA viruses. New York (NY): Oxford University Press; 2009.

[36] Simon-Loriere E, Holmes EC. Why do RNA viruses recombine? Nat. Rev. Microbiol. 2011;9:617–626. 21725337 10.1038/nrmicro2614 PMC3324781

[37] Aljasim TA, Almasoud A, Aljami HA, et al. High rate of circulating MERS-CoV in Dromedary camels at slaughterhouses in Riyadh, 2019. Viruses. 2020;12:1215. 10.3390/v12111215 PMC7692456 33120981

[38] Gao J, Lu G, Qi J, et al. Structure of the fusion core and inhibition of fusion by a heptad repeat peptide derived from the S protein of Middle East respiratory syndrome coronavirus. J Virol. 2013;87:13134–13140. 24067982 10.1128/JVI.02433-13 PMC3838252

[39] Lau SKP, Wong ACP, Lau TCK, et al. Molecular evolution of MERS coronavirus: dromedaries as a recent intermediate host or long-time animal reservoir? Int J Mol Sci. 2017;18:2138. 10.3390/ijms18102138 PMC5666820 29035289

[40] Forni D, Filippi G, Cagliani R, et al. The heptad repeat region is a major selection target in MERS-CoV and related coronaviruses. Sci Rep. 2015;5:14480. 26404138 10.1038/srep14480 PMC4585914

[41] Scobey T, Yount BL, Sims AC, et al. Reverse genetics with a full-length infectious cDNA of the Middle East respiratory syndrome coronavirus. Proc Natl Acad Sci USA. 2013;110:16157–16162. 24043791 10.1073/pnas.1311542110 PMC3791741

