# Supplementary material for: Enhanced replication fitness of MERS-CoV clade B over clade A strains in camelids explains the dominance of clade B strains in the Arabian Peninsula
Source: Emerg Microbes Infect. 2021 Dec 17;11(1):260–74. doi: 10.1080/22221751.2021.2019559 (PMC8812806; doi:10.1080/22221751.2021.2019559)
Supplement: Supplemental Material [file TEMI_A_2019559_SM7135.docx]

# Enhanced replication fitness of MERS-CoV clade B over clade A strains in camelids explain the dominance of clade B strains in the Arabian Peninsula

Nigeer Te^1^, Jordi Rodon^1^, Mónica Pérez^1^, Joaquim Segalés^2,3^, Júlia Vergara-Alert^1,*^, Albert Bensaid^1^

# Affiliations:

^1^IRTA, Centre de Recerca en Sanitat Animal (CReSA, IRTA-UAB), Campus de la UAB, 08193 Bellaterra (Cerdanyola del Vallès), Spain.

^2^UAB, CReSA (IRTA-UAB), Campus de la UAB, 08193 Bellaterra (Cerdanyola del Vallès), Spain.

^3^Departament de Sanitat i Anatomia Animals, Facultat de Veterinaria, UAB, 08193 Bellaterra (Cerdanyola del Vallès), Spain.

^*^Corresponding author: Email: [julia.vergara@irta.cat](mailto:julia.vergara@irta.cat) (J.V.-A.)

# This Supplementary Information file contains:

Supplementary Figures 1-6

Supplementary Tables 1, 2 and 3


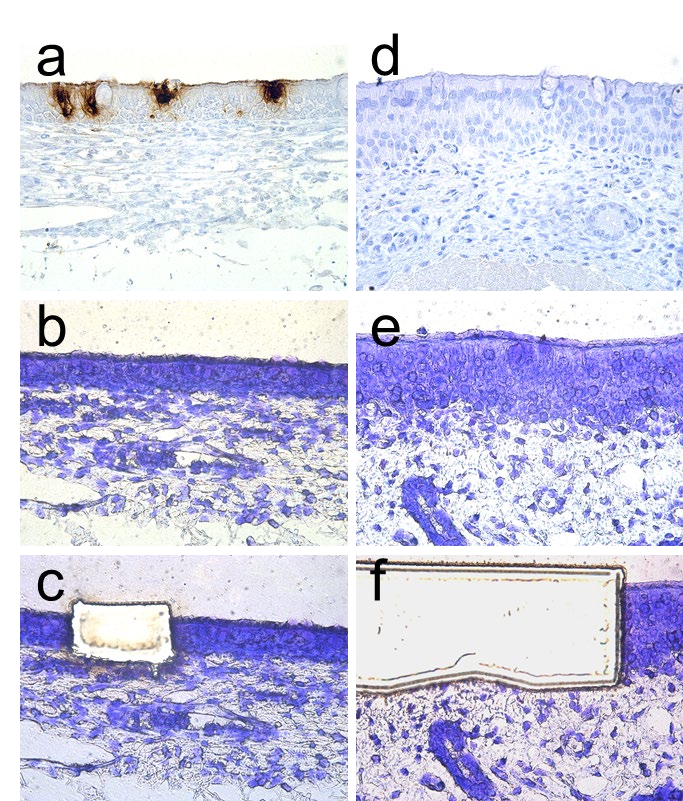


**Supplementary Figure 1. Laser capture microdissection (LCM) of the nasal turbinate mucosa of MERS-CoV EMC/2012 infected alpacas.** For each animal, four consecutive 6-7 μm sections from the same methacarn-fixed paraffin-embedded (MFPE) block were performed and mounted onto Leica RNase-free PEN slides. One of the sections was stained by IHC to detect the presence of the viral N protein and visualize heavily infected mucosa areas (**a**) from non-stained areas apparently devoided of virus (**d**). This IHC stained section served as a template to localize by overlapping on the other contiguous MFPE sections, stained only with cresyl violet, infected (**b**) and "non- infected" (**e**) areas prior LCM. Then LCM was applied to collect the respective selected areas in the mucosa (**c** and **f**).


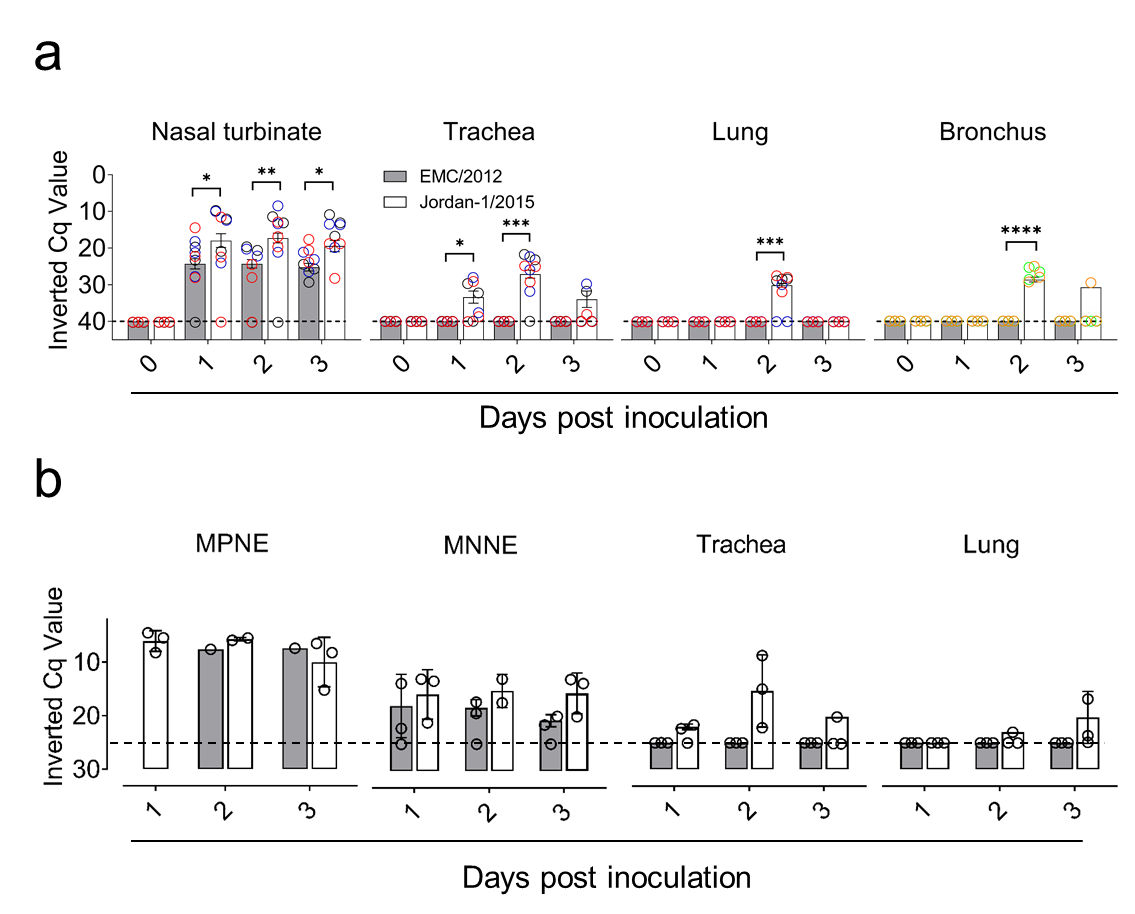


**Supplementary Figure 2. Viral M mRNA loads in respiratory tissues of MERS-CoV EMC/2012 and Jordan-1 infected alpacas.** (**a**) Viral M mRNA loads from respiratory tissues of alpacas obtained on 0, 1, 2 and 3 dpi (1 to 3 dpi, n=3 per day and per strain). Black circles: frontal turbinate, apical trachea and apical lung; blue circles: medial turbinate, medial trachea and medial lung; red circles: caudal turbinate, caudal trachea and caudal lung. Green and yellow circles represent values obtained from large and small bronchus, respectively. Viral loads were determined by real-time RT-qPCR targeting the M mRNA. Each bar represents the mean Cq value ±SEM of infected tissues from 3 animals euthanized on 0, 1, 2 and 3 dpi, respectively. Statistical significance was determined by Tukey's multiple comparisons test. **P* < 0.05; ***P* < 0.01; ****P* < 0.001; *****P* < 0.0001. (**b**) M mRNA loads in MFPE samples. Micro-dissected MFPE nasal epithelia were prepared based on an overlapping template section stained by IHC to localize the MERS-CoV N protein as described in Supplementary Fig. 1. Nasal samples from AP14 sacrificed on 2 dpi could not be processed due to bad preservation. The trachea and lung were scrapped from MFPE tissue sections. RNA extracted from these samples were converted into cDNA and the MERS-CoV M mRNA were determined by a PCR microfluidic assay (Fluidigm Biomark). Error bars indicate SEM when results were positive in more than one animal. Dashed lines depict the detection limit of the assays. Abbreviations: MPNE, MERS-CoV IHC positive nasal epithelia; MNNE, MERS-CoV IHC negative nasal epithelia; MFPE, methacarn-fixed paraffin embedded-tissues; LCM, laser capture microdissection. Cq, quantification cycle. Dashed lines depict the detection limit of the assays.


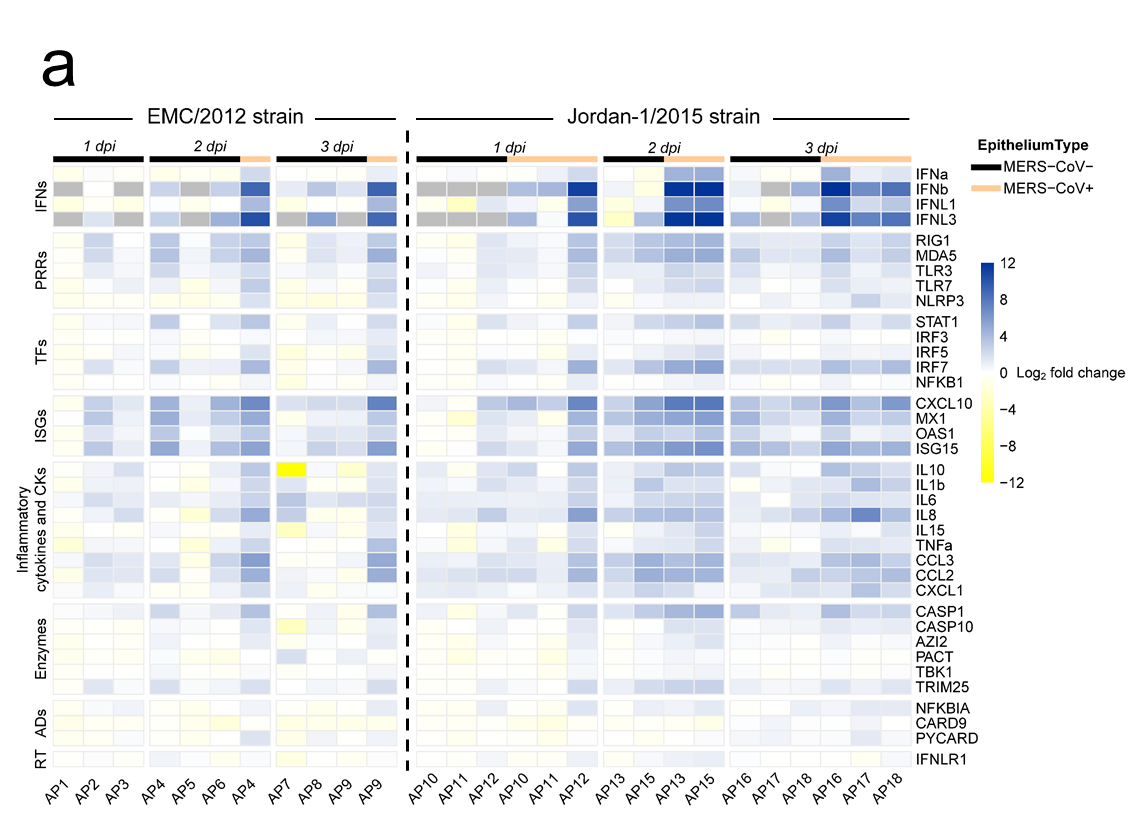


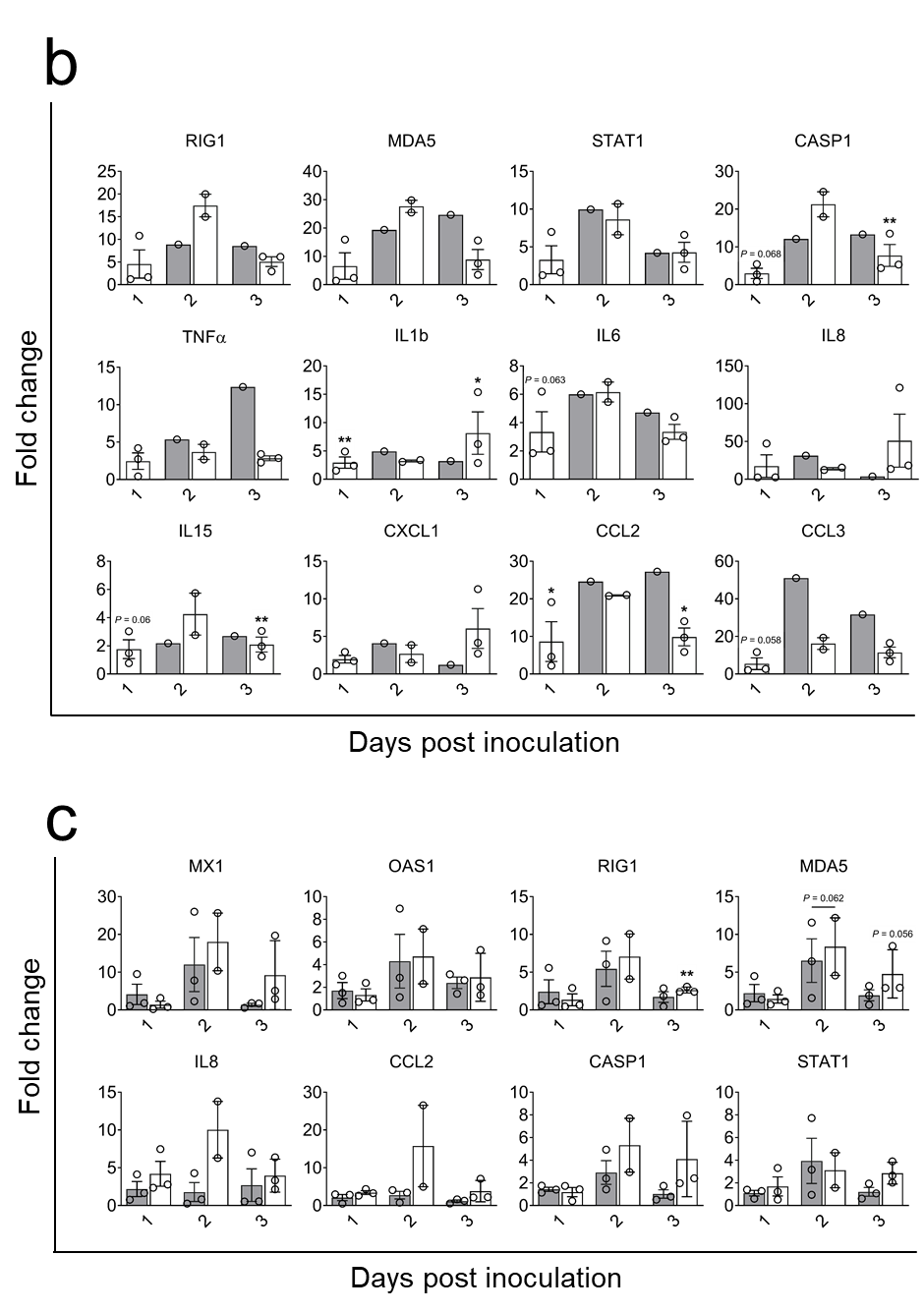


**Supplementary Figure 3. Kinetics of innate immune response genes induced at the nasal epithelia from MERS-CoV EMC/2012 and Jordan-1/2015 inoculated alpacas.** The nasal epithelium of each alpaca (AP1 to AP21, except for AP14) was microdissected

and infected (pink)/non-infected (black) areas, as assessed by IHC, were selected and isolated for RNA extraction and conversion to cDNA. However, we failed to obtain the RNA from AP14 since nasal turbinate of due to the bad preservation. The Fluidigm Biomark microfluidic assay was used to amplify and quantify transcripts of innate immune genes at different dpi (1 to 3 dpi). *HPRT1*, *GAPDH* and *UBC* genes were used as normalizer housekeeping genes and values obtained from the infected animals were compared to those obtained from non-infected alpacas. (**a**) The resulting heatmap shows color variations corresponding to log2 fold change values; blue for increased and yellow for decreased gene expression, respectively. The grey rectangles indicate no expression of the corresponding gene. IFNs, interferons; PRRs, pattern recognition receptors; TFs, transcription factors; ISGs, interferon stimulated genes; CKs, chemokines; ADs, adaptors; RTs, receptors. MERS-CoV+ Epi and MERS-CoV- Epi, MERS-CoV positive and negative epithelium areas as assessed by IHC, respectively. Average fold change of genes in MERS-CoV positive nasal epithelia (**b**) and negative nasal epithelia (***c***) of EMC/2012 (gray bars) and Jordan-1/2015 (white bars) were shown. Data were displayed as means of ± SEM. Statistical significance was determined by Student’s *t*-test. **P* < 0.05; ***P* < 0.01; ****P* <

0.001 (n = 3) compared with non-infected alpacas; #*P* < 0.05; ##*P* < 0.01; ###*P* < 0.001 (n = 3) compared between groups on different dpi.


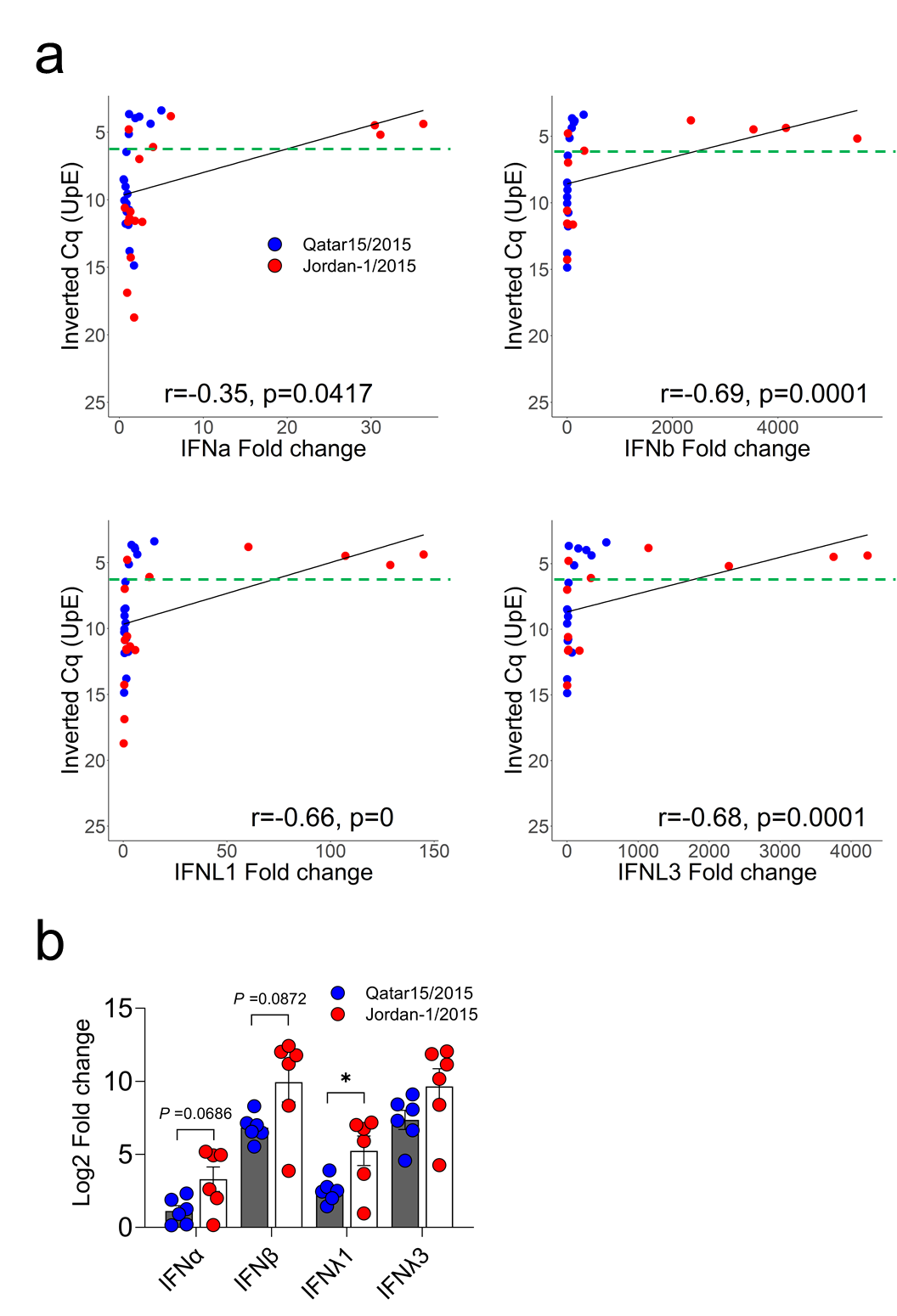


**Supplementary Figure 4. Correlation between MERS-CoV RNA loads and IFN induction in alpaca nasal epithelia.** (**a**) Correlation coefficients of genomic RNA loads (UpE) in the nasal epithelia from alpacas infected with different MERS-CoV strains (Qatar15/2015 and Jordan-1/2015 strains) and IFN expression levels were established using the Spearman’s correlation test. IFNs levels from nasal epithelia of alpacas infected with two strains were normalized against the mean of six non-infected control animals, including three animals from the previous study^1^ and three from the present data. Blue and red spheres represent samples obtained from Qatar15/2015 and Jordan-1/2015 strains infected groups, respectively. The cutoff value of UpE was set to Cq=6.46 (green dashed line) due to the lack of IFNs induction in the samples containing Cq>6.46. (**b**) Average fold change of IFN expression in nasal epithelia (Cq≤6.46) of alpacas infected with Qatar-15/2015 (blue sphere) and Jordan-1/2015 (red sphere). Data were displayed as means of ±SEM. Statistical significance was determined by Student’s *t*-test. **P* < 0.05;

***P* < 0.01; ****P* < 0.001 (n = 6) compared between two groups.


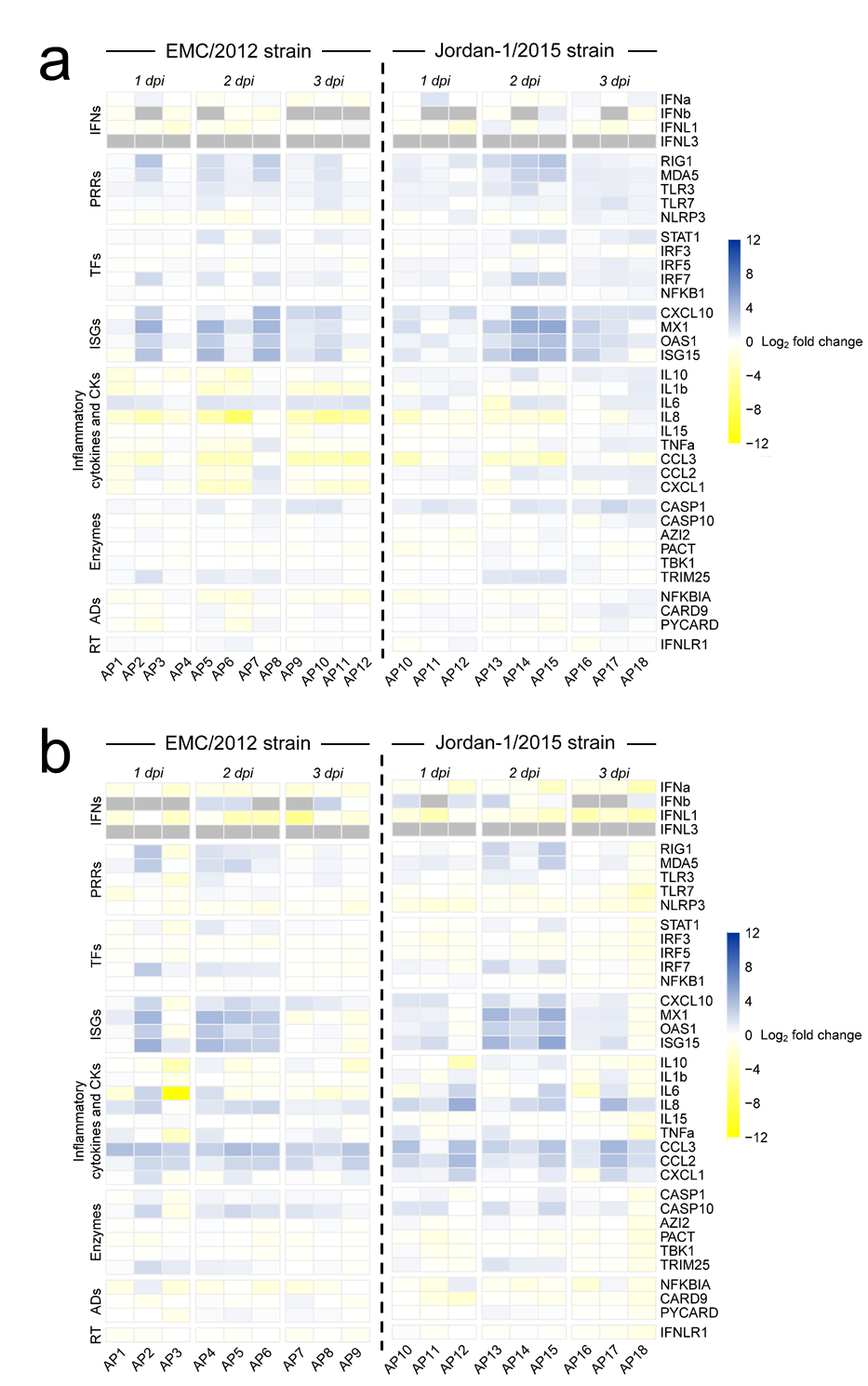


**Supplementary Figure 5. Kinetics of innate immune response genes in the trachea and lung of alpacas upon infection with MERS-CoV EMC/2012 and Jordan-1/2015 strains.** Trachea and lung samples were obtained by scraping MFPE sections from control and infected alpacas (AP1 to AP18). After RNA extraction and conversion to cDNA, a Fluidigm Biomark microfluidic assay was used to amplify and quantify transcripts of innate immune genes at different dpi (1 to 3 dpi). *HPRT1, GAPDH* and *UbC* genes were used as normalizers and values obtained from the infected animals were compared to those obtained from noninfected alpacas. The resulting heatmaps for trachea (**a**) and lung (**b**) show color variations corresponding to log2 fold change values; blue for increased and yellow for decreased gene expression, respectively. IFNs, interferons; PRRs, pattern recognition receptors; TFs, transcription factors; ISGs, interferon stimulated genes; CKs, chemokines; ADs, adaptors.


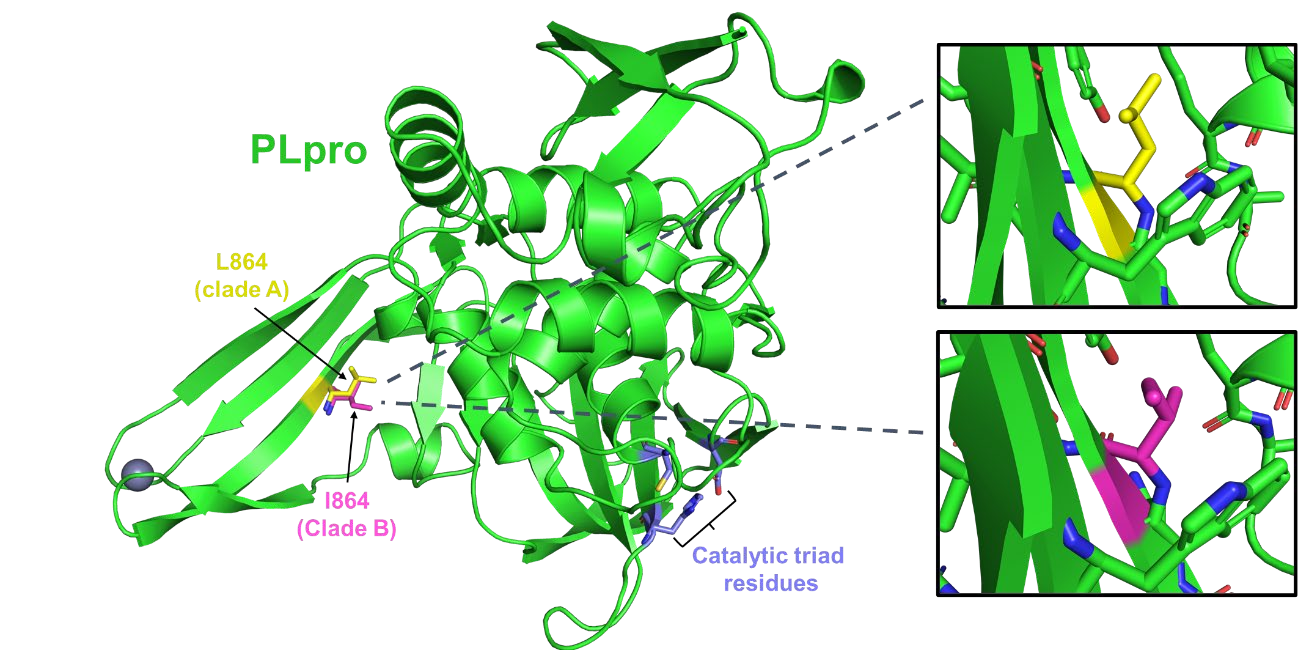


**Supplementary Figure 6. Comparative structural view of the MERS-CoV PLpro nsp3 region in clade A and B strains.** L864 (yellow), I864 (magenta), aa residues in proximity to 1020 (green) and catalytic triad residues (light blue) in PLpro (green helix, PDB ID: 4REZ) are shown as sticks. Neither L864 nor I864 has polar contacts with any nearby aa residues.

# Supplementary Table 1. Sequence and characteristics of primers used for microfluidic qPCR assays in alpacas.

| **Gene category** | **Gene name** | **Primer ID** | **Sequence (5' - 3')** |
| --- | --- | --- | --- |
| Normalizer genes | GAPDH | GAPDH F | GGTCGGAGTGAACGGATTTGG |
|  |  | GAPDH R | TTGAGGTCAATGAAGGGGTCG |
|  | UbC | UbC F | AGGCGAAGATCCAAGACAAGG |
|  |  | UbC R | CCAAGTGCAGAGTGGATTCCT |
|  | HPRT1 | HPRT1 F | CAAAGATGGTCAAGGTCGCAA |
|  |  | HPRT1 R | TCAAATCCAACAAAGTCTGGTCT |
| Interferons | IFNα | IFNα F | TCTTCAGCGAGACACTTGCAA |
|  |  | IFNα R | GTTGGTCAGTGAGAATCATTTCCA |
|  | IFNβ | IFNβ F2 | GCATCCTCCAAATCGCTCTCC |
|  |  | IFNβ R2 | ATGCCAAGTTGCTGCTCCTTT |
|  | IFNλ1 | IFNλ1 F | CTGCCACATGGGCTGGTT |
|  |  | IFNλ1 R | CGATTCTTCCAAGGCATCCTT |
|  | IFNλ3 | IFNλ3 F | CCACCTGGCCCAATTCAA |
|  |  | IFNλ3 R | AGTGACTCTTCAAAGGCGTCCTT |
| Pattern recognition receptors | RIG1 (DDX58) | RIG1 F | ACAAGTCAGAACACAGGAATGA |
|  |  | RIG1 R | CTCTTCCTCTGCCTCTGGTTT |
|  | MDA5 (IFIH1) | MDA5 F | ACACCAGAGTTCAAGAGACTGTAT |
|  |  | MDA5 R | CACCATCATCGTTCCCCAAGA |
|  | TLR3 | TLR3 F | AGAAATAGACAGACAGCCAGAG |
|  |  | TLR3 R | TGCTCCTTTTGATGCTATTAACGA |
|  | TLR7 | TLR7 F | AGAGAGGAGTCACCAGCGTAT |
|  |  | TLR7 R | GACACAAATGCAAATGGAGAC |
|  | NLRP3 | NLRP3 F | ATGGCCACATGGATTTTTGC |
|  |  | NLRP3 R | AAACATTGGCATTGTCCCATTC |

| Transcription factors | STAT1 | STAT1 F | TCTCTGTGTCTGAAGTTCACCCT |
| --- | --- | --- | --- |
|  |  | STAT1 R | GGGAATCACAGGTGGGAAGGA |
|  | IRF3 | IRF3 F | TCACCACGCTACACCCTCTGGT |
|  |  | IRF3 R | GAGGCACATGGGCACAACCTTGA |
|  | IRF5 | IRF5 F | TCAGAAGGGCCAGACCAACACC |
|  |  | IRF5 R | TGCTACGGGCACCACCTGTA |
|  | IRF7 | IRF7 F | CGTGATGTTGCAAGACAACTCA |
|  |  | IRF7 R | TGGTTAACGCCTGGGTCTCT |
|  | NFKB1 | NFKB1 F | GGGACAGTGTCTTACACTTAGCAATC |
|  |  | NFKB1 R | CATCAGAAATCAAGCCAGATGTG |
| Interferon stimulated genes | CXCL10 (IP10) | CXCL10 F | CGTGTTGAGATTATTGCCACAATG |
|  |  | CXCL10 R | GAGGTAGCTTCTCTCTGGTCCT |
|  | MX1 | MX1 F | GAAGATGGTTTATTCTGACTCG |
|  |  | MX1 R | TTCTCCTCGTACTGGCTGT |
|  | OAS1 | OAS1 F | TGAAGAAGCAGCTCGGGAAAC |
|  |  | OAS1 R | AGTAACTGTCTTTTCTGGGCAGC |
|  | ISG15 | ISG15 F | CACAGCCATGGGTGGAATCCTG |
|  |  | ISG15 R | ACAGCATGGAGTCCCTCAGAGTC |
| Inflammatory cytokines and chemokines | IL10 | IL10 F | CTGCTGGAGGACTTTAAGGGT |
|  |  | IL10 R | AGGGGAGAAATCGATGACAGC |
|  | IL1β | IL1β F | AGGATATGAGCCGAGAAGTGGT |
|  |  | IL1β R | CCCTTTCATCACACAAGACAGGT |
|  | IL6 | IL6 F | TCTGGGTTCAATCAGGAGACCT |
|  |  | IL6 R | AGGGGTGCTTACTTCTTCTGGT |
|  | IL8 (CXCL8) | IL8 F | TGTGTGAAGCTGCAGTTCTGT |
|  |  | IL8 R | GCAGACCTCTCTTCCATTGGC |
|  | IL15 | IL15 F | CAGCCTACAGAAGGTCATGAAGTACTC |
|  |  | IL15 R | GGGTAACTCCTTAAGTATCGAAGAAGAG |

|  | TNFα | TNFα F | TGGCCCAGACCCTCAGATCA |
| --- | --- | --- | --- |
|  |  | TNFα R | TTCCAGCTTCACACCATTGGC |
|  | CCL3 (MIP1α) | CCL3 F | GCTCAGCGTCATGCAGGTGCC |
|  |  | CCL3 R | AGCAGGCGGTTGGGGTGTCAG |
|  | CCL2 (MCP1) | CCL2 F | CCAGTAAGAAGATCCCCATGCA |
|  |  | CCL2 R | GTGTGGTCTTGAAGATCACAGCTT |
|  | CXCL1 | CXCL1 F | CGTGCAGGGAATTCACTTCAA |
|  |  | CXCL1 R | GAGAGTGGCTACGACTTCCGTTT |
| Enzymes | CASP1 | CASP1 F | ACTCCACCAAGACCTCAACCAGT |
|  |  | CASP1 R | GGGTAAATCTCCGCTGACTTCTCG |
|  | CASP10 | CASP10 F | CGGTAGCCACGGGAACTGAGTCAT |
|  |  | CASP10 R | ATCTTGCCAGGACCCCTCCGAT |
|  | AZI2 (NAP1) | AZI2 F | TGAGCGTCTCCAGCGCTAA |
|  |  | AZI2 R | CTGCACTTGCGTCACCAGAT |
|  | PACT (PRKRA) | PACT F | TGCAGTTCCTGACCCCTTAATG |
|  |  | PACT R | GATGAATAGCCAGTTCCTGTAGTGAA |
|  | TBK1 | TBK1 F | GTACAGAAAGCAGAAAATGGACCAA |
|  |  | TBK1 R | AACTTGAAGGCCCCGAGAAA |
|  | TRIM25 | TRIM25 F | GCCCGAGCTCCTACAGTATGC |
|  |  | TRIM25 R | GAAGCGACGGTGTAGGTCTTG |
| Adaptors | NFKBIA (IκBα) | NFKBIA F | TCCCTCTTTTCCCCGCAGGTT |
|  |  | NFKBIA R | TGGAGTGGAGTCTGCTGCAGGT |
|  | CARD9 | CARD9 F | GGCAGTGCAAGGTCCTGAAC |
|  |  | CARD9 R | CAGGAGCACACCCACTTTCC |
|  | PYCARD (ASC) | PYCARD F | CAAGCCAGCACCGCACTT |
|  |  | PYCARD R | TCTGTCAGGACCTTCCCATACA |
| Receptors | IFNLR1 | IFNLR1 F | CAGGGTGTGTGATCTGGAAGAG |
|  |  | IFNLR1 R | GTCTGTGTCCAGAGAAATCCAGG |

**Supplementary Table S2. MERS-CoV N protein distribution in alpaca respiratory tracts in response to EMC/2012 and Jordan-1/2015 strains**

|  | 1 dpi | | | | | | 2 dpi | | | | | | 3 dpi | | | | | |
| --- | --- | --- | --- | --- | --- | --- | --- | --- | --- | --- | --- | --- | --- | --- | --- | --- | --- | --- |
|  | **EMC/2012** | | | **Jordan-1/2015** | | | **EMC/2012** | | | **Jordan-1/2015** | | | **EMC/2012** | | | **Jordan-1/2015** | | |
|  | A1 | A2 | A3 | A10 | A11 | A12 | A4 | A5 | A6 | A13 | AP14 | AP15 | A7 | A8 | A9 | A16 | AP17 | A18 |
| Frontal turbinate | － | +/− | － | +/− | － | +/++ | － | － | + | +++ | ++/+++ | +++ | － | － | +/++ | + | + | +/− |
| Medial turbinate | － | +/− | － | +/++ | +/− | +/++ | － | + | + | +++/+  +++ | ++ | +++/+  +++ | － | － | ++ | + | +/− | +/− |
| Caudal turbinate | － | + | － | － | +++/++++ | +/++ | ＋ | － | + | ＋ | ++/+++ | ＋ | － | － | +/++ | + | +/− | ＋ |
| Apical trachea | － | － | － | － | － | +/− | － | － | − | － | +/− | ＋ | － | － | － | － | － | － |
| Medial trachea | － | － | － | － | － | － | － | － | － | － | +/− | ＋ | － | － | － | － | － | － |
| Caudal trachea | － | － | － | － | － | － | － | － | － | － | ＋ | ＋ | － | － | － | － | － | － |
| Large bronchus | － | － | － | － | － | － | － | － | － | － | +/− | +/++ | － | － | － | － | － | － |
| Small bronchus | － | － | － | － | － | － | － | － | － | － | － | +/++ | － | － | － | +/− | － | － |
| Apical lung | － | － | － | － | － | － | － | － | － | － | － | － | － | － | － | +/− | － | － |
| Medial lung | － | － | － | － | － | － | － | － | － | － | － | － | － | － | － | － | － | － |
| Caudal lung | － | － | － | － | － | － | － | － | － | － | － | － | － | － | － | － | － | － |

Abbreviations: dpi, days post inoculation; A, alpaca; -, no positive cells detected; +/-, less than 10 positive cells per tissue section; +, 10 to 50 positive cells per tissue section;

++, 50 to 150 positive cells per tissue section; +++, 150 to 300 positive cells per tissue section; and ++++, more than 300 positive cells per tissue section.

**Supplementary Table S3**. **IFN relative quantification values from nasal epithelia of alpacas used as negative controls of infection.**

| **Sample ID** | **Control negative animal used to study** | **IFNα** | **IFNβ** | **IFNλ1** | **IFNλ3** |
| --- | --- | --- | --- | --- | --- |
| AP13 Epi- | Qatar15/2015^1^ | 0,57459027 | NE | 0,652505894 | NE |
| AP14 Epi- | Qatar15/2015^1^ | 0,47948079 | NE | 0,532744753 | 1,279779162 |
| AP15 Epi- | Qatar15/2015^1^ | 0,90128469 | NE | 1,012091805 | 0,618120869 |
| AP19 Epi- | EMC/2012 and Jordan/2015 (current study) | 0,653389374 | NE | 1,031101821 | 1,102099968 |
| AP20 Epi- | EMC/2012 and  Jordan/2015 (current study) | 2,248673573 | NE | 1,804415044 | NE |
| AP21 Epi- | EMC/2012 and Jordan/2015 (current study) | 1,142581303 | NE | 0,967140683 | NE |

These animals and their gene expression values were further used as normalizer samples to determine gene expression profiles of alpacas infected with clade B MERS-CoV Qatar15/2015^1^ and Jordan-1/2015 strains. Numerical values indicate the individual relative quantification of different IFNs from each non-infected animal normalized against the mean of six non-infected control animals, expressed in fold change values. Abbreviations: AP, alpaca; Epi-, alpaca nasal epithelium with no MERS-CoV infection; NE, not expressed in this tissue.

# References

1. Te, N. *et al.* Type I and III IFNs produced by the nasal epithelia and dimmed inflammation are features of alpacas resolving MERS-CoV infection. *PLOS Pathog.* **17**, e1009229 (2021).

12
